# Protein-Protein Docking with F^2^Dock 2.0 and GB-Rerank

**DOI:** 10.1371/journal.pone.0051307

**Published:** 2013-03-06

**Authors:** Rezaul Chowdhury, Muhibur Rasheed, Donald Keidel, Maysam Moussalem, Arthur Olson, Michel Sanner, Chandrajit Bajaj

**Affiliations:** 1 Department of Computer Science, Institute of Computational Engineering and Sciences, University of Texas at Austin, Austin, Texas, United States of America; 2 The Scripps Research Institute, La Jolla, California, United States of America; University of Michigan, United States of America

## Abstract

**Motivation:**

Computational simulation of protein-protein docking can expedite the process of molecular modeling and drug discovery. This paper reports on our new F^2^ Dock protocol which improves the state of the art in initial stage rigid body exhaustive docking search, scoring and ranking by introducing improvements in the shape-complementarity and electrostatics affinity functions, a new knowledge-based interface propensity term with FFT formulation, a set of novel knowledge-based filters and finally a solvation energy (GBSA) based reranking technique. Our algorithms are based on highly efficient data structures including the dynamic packing grids and octrees which significantly speed up the computations and also provide guaranteed bounds on approximation error.

**Results:**

The improved affinity functions show superior performance compared to their traditional counterparts in finding correct docking poses at higher ranks. We found that the new filters and the GBSA based reranking individually and in combination significantly improve the accuracy of docking predictions with only minor increase in computation time. We compared F^2^ Dock 2.0 with ZDock 3.0.2 and found improvements over it, specifically among 176 complexes in ZLab Benchmark 4.0, F^2^ Dock 2.0 finds a near-native solution as the top prediction for 22 complexes; where ZDock 3.0.2 does so for 13 complexes. F^2^ Dock 2.0 finds a near-native solution within the top 1000 predictions for 106 complexes as opposed to 104 complexes for ZDock 3.0.2. However, there are 17 and 15 complexes where F^2^ Dock 2.0 finds a solution but ZDock 3.0.2 does not and vice versa; which indicates that the two docking protocols can also complement each other.

**Availability:**

The docking protocol has been implemented as a server with a graphical client (TexMol) which allows the user to manage multiple docking jobs, and visualize the docked poses and interfaces. Both the server and client are available for download. Server: http://www.cs.utexas.edu/~bajaj/cvc/software/f2dock.shtml. Client: http://www.cs.utexas.edu/~bajaj/cvc/software/f2dockclient.shtml.

## Introduction

The study of protein-protein interactions plays an important role in understanding the processes of life [1]. Though advancements in X-ray crystallography and other imaging techniques have led to the extraction of near-atomic resolution information for numerous individual proteins; the creation, crystallization and imaging of macromolecular complexes, as extensively required for drug design, still remains a difficult task. Among the atomic structures of proteins deposited in the *Protein Data Bank* [2], only a very small percentage are complexes. Hence, the need for fast and robust computational approaches to reliably predict the structures of protein-protein complexes is growing. An important step towards understanding protein-protein interactions is *protein-protein docking* which can be defined as computationally finding the relative transformation and conformation of two proteins that results in a stable (energetically favorable) complex if one exists.

Given two rigid proteins and some characteristic (e.g., electron density) function(s) of the molecules, one can construct an appropriate representation of them and also define a correlation function based on cumulative overlap of the characteristic functions. Then it is possible to conduct a combinatorial search in a 6D parameter space of all possible relative translations and orientations of the two proteins to find the optimal. Hence in computational perspective, docking is a search over the space of possible orientation of two proteins to find the (set of) optimum(s) of a scoring function designed to mimic physico-chemical interaction of proteins.

The combinatorics of the search can be reduced by using coarse grids and rotational angles [3], and by using a-priori knowledge of suitable binding sites [4]. For docking without prior knowledge about possible binding sites, exhaustive sampling is required to improve the probability of finding the global minimum energy configuration. In such cases, Fast Fourier Transforms has been used to speed up the cumulative scoring function computations [3]–[5]. Spherical Fourier correlation based approaches were presented in multiple studies [5]–[9]. However, if binding sites are known, or inferred based on some initial stage docking, then a finer resolution search involving local flexibility can be applied to improve the accuracy of the fit [10]–[12].

Accuracy of docking predictions is dependent on the scoring model’s ability to distinguish between native and non-native poses. Docking based on structural (shape) complementarity alone has shown to be adequate for a range of proteins [4], [13], [14]. To represent shape complementarity, a grid based double skin layer approach became the base of many variations and software, e.g., DOT [15], ZDOCK [16], PIPER [17], MolFit [18], [19] and RDOCK [20]. However, energy and bioinformatics based scoring terms have been shown to improve the accuracy of predictions and a combination of multiple scoring terms have become the norm in current docking software. For example, DOT 2.0 [15] is based on van der Waals energy and Poisson-Boltzmann electrostatics, ZDock 3.0.2 [21] uses pairwise shape complementarity, electrostatics, and pairwise potentials known as Interface Atomic Contact Energies (IFACE), PIPER [17] is based on shape complementarity and electrostatics using a Generalized Born (GB) type formulation, and uses a new class of structure-based potentials referred to as DARS (Decoys As the Reference State) where the potentials are derived from a large set of docking conformations as decoys. FRODOCK [22] is a recent spherical harmonics based docking tool that uses van der Waals, electrostatics and desolvation potential terms in its correlation function. Some docking or reranking techniques solely use coarse-grained potentials trained on large benchmark of decoys [23], [24]. We leave the reader to consult the reviews [25]–[29] for further information.

In [30] we described a non-equispaced Fast Fourier Transform (NFFT) based rigid-body protein-protein docking algorithm for efficiently performing the initial docking search (based on shape and electrostatics complementarity). Compared to traditional grid based Fourier docking algorithms, the algorithm was shown to have lower computational complexity and memory requirement. The algorithm was extended in [31] to F^2^ Dock (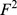  =  Fast Fourier), which included an adaptive search phase (both translational and rotational) for faster execution.

In this paper we describe a new version (F^2^ Dock 2.0 ) which includes improved shape-complementarity and electrostatics functions as well as a new on-the-fly affinity function based on interface propensity and hydrophobicity. The current version uses uniform FFT, but exploits the sparsity of FFT grids for faster execution and restricts its search within a narrow band around the larger molecule. A clustering phase penalizes docking poses that are structurally similar to poses with better scores and a set of efficient on-the-fly filters penalize potential false positives based on Lennard-Jones potential, steric clashes, interface propensity, interface area, residue-residue contact preferences, antibody active sites, and glycine richness at the interface for enzymes. The filters are implemented using fast multipole type recursive spatial decomposition techniques [32], [33]. A solvation energy based reranking program GB-rerank [32], [34] has also been implemented using an approximation scheme which can be tuned for speed-accuracy trade-off. Both F^2^ Dock 2.0 and GB-rerank have been implemented as multithreaded programs for faster execution on multicore machines. Our molecular visualization software *Te
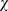
Mol* serves as a front-end to F^2^ Dock 2.0 in a client-server mode of execution [35]. *F* ^2^ *Dock* has been calibrated based on an extensive experimental study of the rigid-body complexes from Zlab benchmark 2.0 [36] and tested on Zlab benchmark 4.0 [37] (which includes the complexes from benchmark 2.0).

The paper is organized as follows. In the next section, we describe the latest version of F^2^ Dock 2.0. Results are presented and discussed in the next section, followed by conclusions and plans for future research.

## Methods

Let 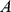 and 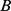 be two proteins with 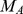 and 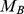 atoms respectively. Without loss of generality, we assume that 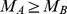, i.e., 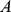 is the larger of the two proteins. We refer to 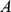 and 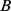 as “receptor” and “ligand”, respectively.

**Figure 1:**
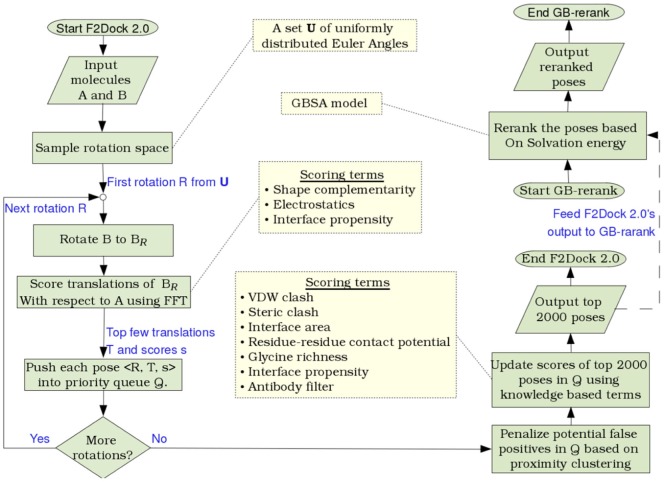
High-level overview of rigid-body protein-protein docking using F^2^ Dock 2.0 and GB-rerank. F^2^ Dock 2.0 performs exhaustive 6D search in discretized rotational and translational space where it computes a score for each sampled orientation of the ligand with respect to a stationary receptor. The scoring function is a weighted combination of shape complementarity, electrostatics and interface propensity based affinity terms (refer to Section 2.3 for details). The top few orientations (poses) of the ligand are kept in a priority queue. Then top several thousand poses from the queue are clustered based on the distance between the geometric centers of different poses of 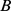. All but the best scoring pose of a cluster is penalized by reducing the score. The resulting reordered list is then passed through several soft filters in order to further penalize potential false positives. Finally, as a separate post-processing step, the ranked docking poses are re-scored and reranked based on the change in solvation energy caused by each pose.

Figure 1 gives a high level overview of the algorithm. The rest of this section details the various aspects of the algorithm.

### 2.1 Overall Strategy

First, F^2^ Dock 2.0 performs exhaustive search in 6D space of relative configuration of 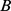 with respect to 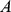. We use a discrete and uniform sampling of 3D rotational space and then use FFT to score a discrete 3D translational space. Given 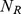 rotational samples and 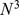 translational grid, F^2^ Dock 2.0 computes 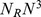 scores. However, only a constant multiple of 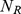 scores and their corresponding poses are retained for the next step. Let us denote this set as 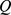. A particular pose is expressed as a tuple 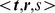 where 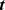 is the translation, 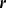 is the rotation and 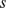 is the corresponding score.

We apply a very simple clustering scheme based on proximity of the poses in 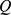 to reshuffle the order such that the top few poses are dissimilar to each other. Though this step does not affect the overall ratio of true and false positives, it increases the chance of finding at least one near-native solution at the top of the order. It is important because the next stage of filtering is only performed on the top few (2000 by default) poses. Let this reduced list be called 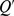.

The filters are designed based on knowledge-based scoring potentials, which are described in Section 2.5, to update the scores of the poses of 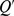, reorder them and output them as final predictions from F^2^ Dock 2.0. Some filters are defined only for specific types of proteins like Antibodies or Enzymes.

The results from F^2^ Dock 2.0, or a subset of it, can optionally be reranked using a solvation energy (generalized Boltzman model) based reranker called GB-rerank which generally improves ranks of near native solutions.

### 2.2 Exhaustive Search Using FFT

#### 2.2.1 Rotational sampling

The rotation space is sampled using uniformly distributed Euler angles as in [13], [15], [38]. For each sampling interval 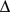 the sample set is equivalent to a set of points uniformly distributed on a projective sphere such that the angular distance [39] between any two points in the set is at most 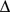. This approach provides a much better distribution of samples than sampling each angle (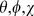) separately and requires fewer samples for the same 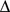.

#### 2.2.2 Translational sampling and scoring

FFT-based scoring of the translational grid (see, e.g., [3], [4]) involves two forward (one each for molecules 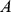 and 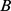) and one inverse FFT computations. Since the forward FFT of the stationary molecule 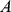 can be precomputed, in practice, only one forward (involving molecule 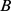) and one inverse FFT must be computed for each rotation. Current version of F^2^ Dock 2.0 uses uniform FFT but exploits the sparsity of the input and the output grids for faster computation as follows.

- Forward FFT (sparsity of the input grid): The input grids are large enough so that they can at least contain the following two spheres side-by-side: the smallest sphere enclosing molecule 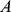, and another sphere having the same radius 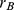 as the largest distance from the geometric center of molecule 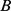 to an atom of 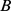. Hence, when discretized to such a grid molecule 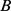 will occupy only a fraction of the grid points around the grid center. Thus many grid planes will remain completely empty (i.e., have zero values only). 3D FFT is sped up by ignoring recursive calls that compute 2D FFT’s of such empty planes.
- Inverse FFT (sparsity of the output grid): In the output translational grid we need to score only the grid points that are within a band around the stationary molecule 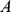 such that if the geometric center of molecule 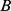 lies outside the band the two molecules can never touch. Note that the number of gridpoints in the band is 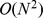 as opposed to 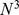. This band can be approximated during the initial precomputation phase by running a sphere of radius 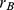 (defined in previous paragraph) over the surface of 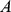 which can be done using a single call to FFT. This sparseness of the output grid is exploited to speed up inverse FFT.

#### 2.2.3 Cost of FFT-based affinity function computations

For any rotation 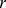 the FFT-based scoring takes 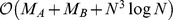 time, where 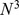 is the size of the FFT grid. Hence, for 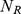 rotations the total running time is 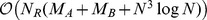. We use the publicly available FFTW package [40] for our FFT-based scoring.

### 2.3 FFT-based Affinity Functions

F^2^ Dock 2.0 uses three affinity functions during exhaustive search over the rotational and translational space. The score is defined as a weighted sum of the shape complementarity, electrostatics and interface propensity scores. Using a new approach to skin-core definition and weighting, the shape complementarity term has been greatly improved from the original version (*F* ^2^ *Dock* ), and the interface propensity is a novel addition defined using statistical residue potentials.

#### 2.3.1 Shape complementarity (SC)

**Figure 2:**
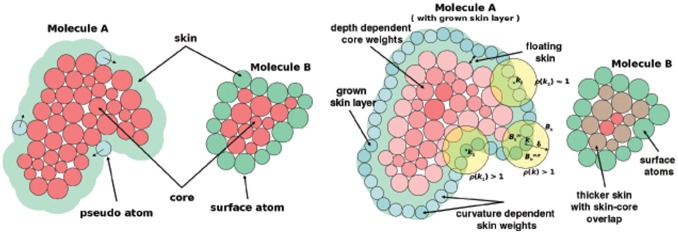
Definition of skin and core for shape complementarity. (Left) Traditional *double skin-layer* approach for shape complementarity, (Right) Improved approach with curvature-based weighting of skin atoms and depth dependent weighting of core atoms of molecule 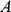, and depth dependent weighting of the atoms of 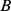.

The original version of F^2^ Dock [31] used the traditional double skin layer approach for shape complementarity [41]. Two *skin regions* are defined (Figure 2): a *grown skin region* around 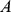, and the *surface skin* of 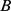, which consists of the surface atoms of 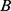. The atoms of 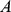 and the inner atoms of 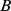 form *core regions*.

A good docking pose of 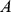 and 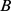 will have large skin-skin overlap and small core-core overlap, and in order to identify such poses constant positive imaginary weights are assigned to the core atoms and constant positive real weights to skin atoms/pseudo-atoms. An integral of the superposition of the molecules has two real contributions: the core-core overlaps contribute negatively and the skin-skin overlaps contribute positively. Hence the real part can be used to rank docking poses based on shape complementarity. The magnitude of the imaginary part of the integral due to skin-core clashes (caused by pseudo-atom vs atom overlaps) is not desirable and assigned a smaller negative weight in the accumulated score.

#### Improved double skin approach

The current version (F^2^ Dock 2.0 ) uses an improved double skin layer approach which differs from the traditional approach in four ways. First, the receptor skin layer does not touch the receptor van der Waals surface and the radius of skin atoms are different. This is based on our observation of the gap between the VDW surfaces of the receptors and ligands in Zlab benchmark 2.0 [36]. Second, the weights assigned to receptor skin atoms are computed based on the curvature of the skin around that atom. Such weighting encourages convex-concave and concave-convex interfaces as opposed to large flat interfaces. Third, the core atoms of molecule 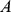 are assigned weights using an increasing function of depth (distance of the atom center from the surface of 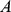). Such weightings discourage deeper core-core overlaps more strongly. And fourth, since in the traditional approach the ligand skin is defined using its surface atoms, the skin thickness varies and can be too thin in some areas. Therefore, we use a double layer of ligand atoms as its skin. Refer to supplemental materials (Supplement S1) for in depth discussion on the skin-core definition as well as FFT based correlations.

#### 2.3.2 Electrostatics (E)

In the previous version (F^2^ Dock ) [31], we defined the electrostatics affinity function similar to the simplified model for electrostatics described by Gabb et. al. [4], which allows efficient FFT-based computation. The first protein’s electric potential is computed and matched against the charges in the other. In this version (F^2^ Dock 2.0 ), we replace point charges with a Gaussian to reduce discretization errors on the grid (See Supplement S1 for details).

#### 2.3.3 Interface propensity (IP) and hydrophobicity (HP)

F^2^ Dock 2.0 scores the interfaces between molecules 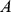 and 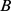 using the per-residue interface propensity values computed in [42] which are based on relative frequencies of residues in the interfaces of a set of 63 protein-protein complexes [43]. Let 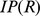 denote the natural logarithm of the interface propensity value of a residue 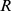. The 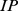 values for the 20 amino acid residues lie between −0.38 (ASP) and 0.83 (TRP). A residue with higher 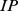 value is likely to occur more frequently in a protein-protein interface than one with a lower value.

Let 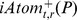 and 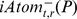 denote the set of atoms in the interface of 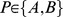 in this docking pose that have positive and negative 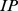 values, respectively. Also let 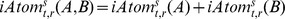, for 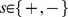. We assign an interface propensity score to the pose:
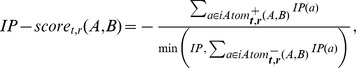
where 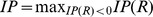. We also penalize very small interfaces by setting 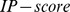 to a negative value when 
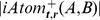
 is below a user-defined threshold.

See Suppplement S1 for details on the FFT based formulation and parameter selections.

### 2.4 Proximity Clustering

We consider all docking poses in 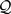 sorted by score, and penalize a pose if there are similar poses with higher score. Suppose 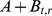 is a docking pose with score 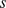 that is currently being considered. For a given 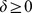, let 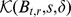 be the number of docking poses with score at least 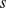 and with geometric center within distance 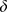 from that of 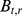. Then we penalize 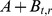 as follows.

1. If 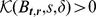, then the score of the pose 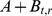 is reduced by 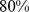,
2. otherwise, if 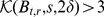, then the score of the pose 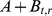 is reduced by 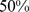,
3. otherwise, if 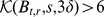, then the score of the pose 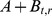 is reduced by 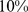.

The objective of this proximity based penalty is to increase diversity at the top of the order which increases the possibility of getting at least one near native solution at the top of the order. But it also penalizes all true positives (except one). As a result, F^2^ Dock 2.0 tends to get a near native solution at high ranks for many complexes, but the total number of near native solutions for any particular complex is not high.

#### 2.4.1 Cost of clustering

We use our dynamic packing grid data structure [33] to speed-up this computation, and the overall time required for this step is 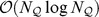 (with high probability), where 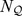 is the number of poses originally in 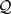.

### 2.5 Filters

To penalize potential false positives and thus improve the ranks of correct solutions, F^2^ Dock 2.0 uses several filters based on the Lennard-Jones potential, the number of steric clashes, interface area, interface propensity, residue-residue contact preferences, antibody active sites, and the frequency of glycine residues at the interface for enzymes.

Only the interface regions of the two molecules at a given pose contribute to the terms used in the filters. We have developed an octree-based hierarchical spatial decomposition technique [32] and the Dynamic Packing Grids data structure [33] for efficiently locating the interface regions and for computing local interactions. Since the overall algorithm for computing each term is similar and only varies in the exact type of local interactions we compute, we present the algorithm only once (in our discussion of the interface propensity filter).

#### 2.5.1 Interface propensity filter

This filter computes the interface propensity of the interfaces of the molecules at a given pose and penalizes or rewards the pose based on empirically determined thresholds.

We sample and weigh quadrature/integration points from the surface of each molecule as described in [32], [34]. The sampling can be considered a decomposition of the surface into small patches, where each quadrature point is representative of a patch 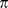. The weight of a quadrature point is the same as the area 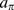 of the corresponding patch. Each quadrature point is also labeled with the average of the interface propensity values of the atoms near the point. Let, 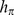 denote the interface propensity label of a quadrature point. The interface propensity contribution of a quadrature point is defined as 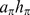 if 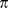 is on the interface, and 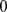 otherwise.

We approximate the interface propensity as 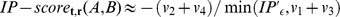, where, 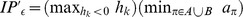 (defined in Section 2.3.3). Here 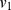 and 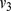 are the sum of the negative interface propensity contributions of the quadrature points of two molecules; and 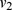 and 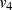 are the sum of the positive contributions. We reward docking poses with large 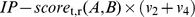 values, and penalize a pose if its 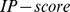 is below a threshold.

The crucial step in the approximation is identifying quadrature points that are on the interface. We store the quadrature points into a DPG data structure, and we also store them in an octree. The octree is a hierarchical and adaptive subdivision of space such that a node of the tree represents a regular cube in 3-space, A node is split if it contains more than a user-defined number of quadrature points in it. Given a specific pose, we trace the two octrees starting from the roots to identify the leaves that are close to each other. Then for each pair of neighboring octree-cells, we use DPG to identify quadrature points in one leaf which have a neighbor in the other leaf. The overall cost of the algorithm is 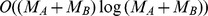 with high probability, where 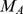 and 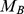 are the number of atoms in 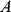 and 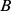. However, in practice it runs even faster and approaches 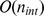, where 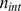 is the number of quadrature patches on the interface.

#### 2.5.2 Residue-Residue contact filter

Contact preferences derived from a non-redundant set of 621 protein-protein interfaces of known high resolution structures [44] are used to penalize potential false positives. Two residues are considered to be in contact if the distance between their 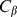 atoms (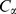 for Gly) is less than 6 Å. In [44], log-normalized contact preferences 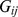 for each pair of amino acid types are reported. Positive values of 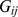 indicate that residues 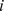 and 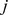 prefer to form contacts, negative values indicate the opposite.

Given a docking pose 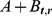, we identify all residue-residue contacts at the interface of the two molecules using a fast algorithm similar to the one used in Section 2.5.1, and compute the sum of all positive and negative 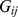 values denoted by 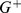 and 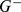, respectively. Then we penalize the pose if the ratio of 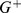 and 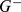 is outside a user-specified range.

#### 2.5.3 Lennard-Jones filter

We approximate the Lennard-Jones (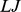) potential between molecules 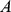 and 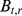 as follows: 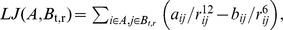 where 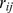 is the distance between atoms 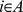 and 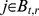, and constants 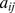 and 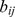 depend on the atom types. The well depths 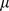 and equivalence contact distances of homogeneous pairs 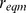 are taken from the Amber force field [45], [46]. Poses with positive 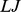 potential are penalized. However, we allow soft clashes in the cases of unbound-(un)bound docking by reducing the 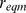 values by a constant factor which effectively reduces the inter-atomic clash distances (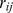 values).

#### 2.5.4 Clash filter

Two atoms 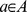 and 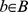 with van der Waals radii 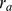 and 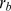, respectively, are considered to be clashing provided the distance between their centers is smaller than a threshold. F^2^ Dock 2.0 counts the total number of clashes 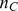 between molecules 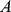 and 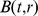 and penalizes if 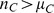, where 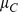 is a user-defined constant.

#### 2.5.5 Interface area filter

This filter penalizes a docking pose if the interface area is outside the range of areas derived empirically from known native interfaces. We define the interface area as the sum of the weights of the quadrature points on the interface, where the weights and the interface is defined the same way as in the interface propensity filter.

#### 2.5.6 Glycine filter

Enzyme active sites are rich in Glycines, particularly *G-X-Y* and *Y-X-G* oligopeptides, where 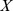 and 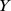 are polar and non-polar residues, respectively, and 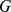 is glycine [47]. The 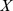 and 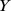 residues are typically small in size and low in polarity, and the frequency of those two types of oligopeptides is significantly higher in enzyme active regions than in other parts of the enzyme molecule. Therefore, enzyme surface oligopeptides with these properties are marked and for a given docking pose, the number of these motifs occurring at the interface are counted. If this count is below a user-specified threshold 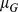, the pose is penalized. Conversely, poses with higher *G-X-Y*/*Y-X-G* frequency at the interface are rewarded by adding this (weighted) count to the total score.

#### 2.5.7 Antibody-Antigen contact filter

As reported in [48] (available at http://www.bioinf.org.uk/abs/allContacts.html) based on a set of 26 known antibody-antigen complexes, each of the following three regions in an antibody will make at least one antigen contact (burial by at least 1 Å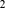 change in solvent accessibility): 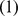 CDR-L1 or CDR-H1, 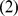 CDR-L3 and 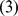 CDR-H3.

Given a potential antibody-antigen docking pose, F^2^ Dock 2.0 computes 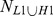, 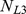 and 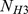, the number of antigen atoms that are in the close neighborhood of any atom in the antibody regions CDR-L1/CDR-H1, CDR-L3 and CDR-H3, respectively. The CDR (Complementarity Determining Region) loops are identified using the method described in [49]. F^2^ Dock 2.0 penalizes poses if the computed values are outside the ranges observed in the native antibody-antigen interfaces in our training set.

#### 2.5.8 Cost of filtering

Using our algorithm described in [32] based on octrees [50] and our *Dynamic Packing Grid* (DPG) data structure [33], the scores for each filter can be evaluated in 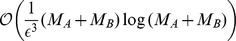 w.h.p. (for an input of size 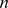, an event E occurs w.h.p. (with high probability) if, for any 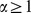 and 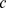 independent of 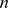, 
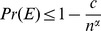
.) time and 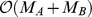 space. Assuming that each filter is applied on at most 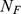 configurations, the total time taken by all filters is 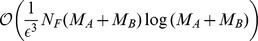 (w.h.p.). Assuming 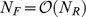, where 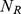 is the number of samples in the rotations space, the running time reduces to 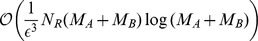 (w.h.p.).

### 2.6 Solvation Energy Based Reranking with GB-rerank

GB-rerank approximates the change in solvation energy of a complex and reranks the list of top docking poses produced by F^2^ Dock 2.0 based on the resulting 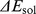 values. In order to approximate 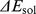, GB-rerank precomputes the 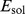 values for molecules 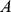 and 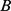, and then computes 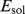 for each docking pose.

The solvation energy 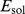 consists of the energy to form cavity in the solvent (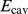), the solute-solvent van der Waals interaction energy (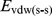), and the electrostatic potential energy change due to the solvation (also known as the polarization energy, 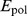) [51]–[55].
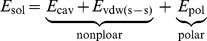



The first two terms are often modeled as 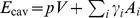 and 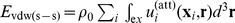, where 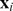 and 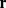 are the position and center of atom 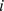, 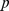 is the solvent pressure, 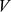 is the molecular volume, 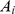 is the solvent accessible surface area of atom 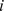 and 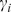 is its solvation parameter, 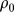 is the bulk density, and 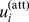 is the van der Waals dispersive component of the interaction between atom 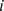 and the solvent [51], [56]. The last term, 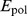, can be approximated using the Generalized Born theory [57], whereby:
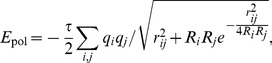
(1)where 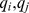 are the atomic charges and 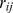 is the distance between atoms 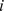 and 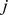, 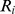 is the effective Born radius of atom 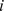 and 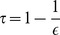.

The algorithms for rapidly approximating these terms have been presented in [32].

#### 2.6.1 Overall cost of reranking

Assuming that GB-rerank is applied on 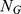 docking poses, its total running time is
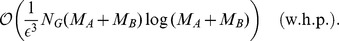



Typically 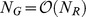, where 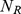 is the number of samples in the rotations space, and so the running time reduces to 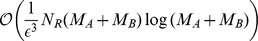 (w.h.p.).

### 2.7 Dataset Preparation

F2Dock takes two PDB files as inputs. First the PDB files are processed by PDB2PQR [58] where missing atoms such as Hydrogens are added, the protein is optimized for hydrogen bonding, and charge and radius parameters are assigned using the AMBER force-field available in PDB2PQR. If the given PDB has missing residues or too many residues with missing backbone atoms, then our curation process fails and F2Dock cannot be used without manually curating the PDB or using other curation software.

Then pseudo-atoms are added above the surface of the receptor (i.e., stationary molecule), and surface atoms of the ligand (i.e., moving molecule) are detected. These atoms are marked as skin atoms, and the rest as core atoms.

### 2.8 Parameter Selection Based on Complex Type

F^2^ Dock 2.0 has several free parameters in its pipeline. We can broadly classify the parameters into several groups. For parameters like the charge and radii of atoms, or the hydrophobicity and interface propensity of residues etc., we either use well-established parameters (for example, from the AMBER [59] force field) or derive from previously published results (for example, interface propensity values from [42]). Some parameters are internal to a scoring function for example the distance dependent dielectric for electrostatics, or the thickness of the skin used in shape complementarity. These parameters are trained using manual parameter sweeps based on a small number (4–5 per complex type) of complexes. However, we produced multiple configurations for each complex and chose the set of parameters which maximizes the corresponding individual scoring term for the near native poses. A similar strategy was used for selecting the thresholds used to penalize poses during filtering. Finally, there are the parameters that govern the weights assigned to different scoring terms when they are combined as well as the weights (or percentages) by which poses are penalized. These parameters are the most difficult to train as the scoring terms are not independent and the relative influence of a term might vary for different complexes. These parameters were trained based on the 60 complexes from Zlab’s protein-protein benchmark 2.0 [36] as follows.

The complexes in the benchmark are categorized into four main types: Antibody-Antigen (A) and Antibody-bound Antigen (AB), Enzyme-Inhibitor/Enzyme-Substrate (E), and other (O) types. We identify that the classification is not only functional, but it also has significant effect on scoring function design since different scoring terms bear different level of significance for different categories of complexes. For example, it is known that binding interfaces of Enzymes are rich in Glycines, which lead us to design a filter based on Glycine richness and it is applied only for Enzyme type of complexes. For each class of complexes (9 Antibody-Antigen, 9 Antibody-bound Antigen, 21 Enzyme-Inhibitor/Enzyme-Substrate and 21 Others), we train the weight parameters separately. The objective for the training is to improve the ranks of near-native solutions for as many complexes as possible. We performed parameter sweeps for each of the weights that combines the FFT based scores based on the above objective for each of the categories. Then we examined the effect of applying each of the filter, one at a time, and controlled its penalty to improve the results.

We do realize that our manual scheme has its drawbacks, specially since it does not sufficiently cover the entire space of possible values for the parameters. We are actively trying to use machine learning schemes to train the parameters in a more robust way. However, so far our attempt of using quadratic programming and random forest learning based on thousands of negative and positive examples based on this benchmark have failed to produce a set of parameters which outperform the manually calibrated set of parameters.

Default values of all the parameters for different types of complexes can be found in the user manual for F^2^ Dock 2.0 downloadable from our website (link given in the abstract).

#### 2.8.1 Automated detection of complex types

Since F^2^ Dock 2.0’s parameters are optimized separately for antibody-antigens and enzyme-inhibitors/enzyme-substrates, and a general set of parameters are used for all other types of complexes, the user only needs to specify the complex type to ensure the set of optimized parameters are applied. If the type is unknown, F^2^ Dock 2.0 tries to determine which set of parameters to use as follows. If F^2^ Dock 2.0 locates the six CDR loops (L1, L2, L3, H1, H2 and H3) in the protein sequence using the algorithm in [49], it identifies it as an antibody and uses the corresponding parameter set. Otherwise, if neither molecule is identified as an antibody and at least one of the molecules has at least 200 residues and at least 8% of its surface residues are Glycines then F^2^ Dock 2.0 uses the enzyme complex parameter set. Finally, if both tests fail, a set of parameters for the general case is used. Among the complexes in the Zlab benchmark 2.0, F^2^ Dock 2.0 fails to identify only one antibody (1KXQ) and three enzymes (1AY7, 1UDI and 2MTA). See Supplement S1 for details.

## Results

We present the results of our experiments to explore the contribution of the new scoring terms and filters available in F^2^ Dock 2.0 as well as the solvation energy based re-ranker GB-rerank on prediction accuracy. These experiments are carried out on the set of complexes in Zlab’s benchmark 2.0 [36] which contains 60 complexes. Then we run F^2^ Dock 2.0 with the best set of parameters on the complexes in the Zlab benchmark 4.0 [21], and compare the performance with ZDock 3.0.2 [37]. The complexes in both the benchmarks are categorized into rigid-body (easy), medium and difficult (flexible) based on the RMSD between the bound and unbound states of the proteins. They are also categorized into four main types: Antibody-Antigen (A) and Antibody-bound Antigen (AB), Enzyme-Inhibitor/Enzyme-Substrate (E), and other (O) types. As mentioned before, F^2^ Dock 2.0 uses different set of parameters for the different categories and we have also compared our results for each category separately.

### 3.9 Evaluation Criteria

F^2^ Dock 2.0’s search leaves the receptor stationary and searches over the orientations of the ligand. Hence, to evaluate the accuracy of a predicted pose, we compute the deviation between the predicted position of the ligand and its correct position as the root mean squared distance (RMSD) of the interface atoms. Note that correct position of the ligand for unbound test cases can be approximated by aligning the unbound components to their bound counterparts. The unbound ligands in the ZLab benchmarks are provided after alignment with bound counterparts and hence can be used as the approximate truth without further manipulations. We assume that an atom is on the interface if the distance between its center and the center of any atom on the other molecule is less than 10Å. We define 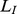 as the set of all backbone atoms of the ligand which are on the interface when the ligand is placed in its native pose w.r.t the receptor (to find the native pose for an unbound case, we simply align the unbound receptor and unbound ligand to their bound counterparts). If the position of ligand atom 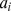 is 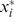 in the native pose and 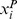 in a predicted pose 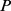, then the interface RMSD is computed as 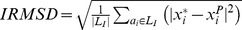. A predicted solution is considered a *hit* provided its IRMSD value is at most 5Å.

In the remaining text and supplement S1, we refer to the hit with the lowest RMSD as the ‘best’ hit and the hit with the highest rank as the ‘top’ hit. In most of our results, we compare protocols based on the rank of the ‘top’ hit. Given a set of complexes 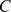, and a protocol 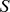, we define 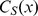 as the set of complexes such that for each complex 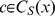, the top hit lies within the range 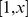. Clearly, for a given 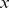 a higher 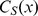 is better. Hence, to compare the accuracy of two protocols 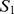 and 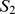, we can simply compare 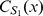 and 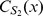 for different 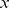. In general we use a few specific values for 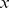 ([1,1], [1], [5], [1], [10], [1], [50], [1,100], [1,500] and [1,1000]). We are specially interested in the first few ranges which shows off the accuracy of the scoring model, and the last range which shows off the applicability of the model over a broad range of complexes.

Two residues 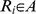 and 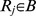 are considered to be in contact if the distance between the centers of any atom 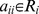 and any atom 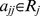 is less than a threshold. The set of residue-residue contacts for the native pose of the receptor and ligand are defined as the native contacts 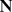. For a given predicted pose, we compute the set of residue-residue contacts for that pose as 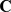. The set of native contacts for that pose is hence defined as 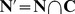. Now, we define another metric based on native contacts as 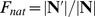. We follow the well known CAPRI criteria that uses a combination of 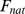 and 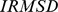 to classify predictions as high, medium, acceptable and incorrect.

### 3.10 Analyzing the Improvements due to New Affinity Functions and Filters

#### 3.10.1 Effectiveness of the new skin-core definition

We have compared the new improved double skin approach to the traditional approach (used in F^2^ Dock [31]) in terms of their prediction accuracy on the rigid-body complexes of the Zlab Benchmark 4.0. In these tests only the shape complementarity term was used, and hence the results are not as accurate as the default combination of scoring and filtering terms can produce.

**Figure 3:**
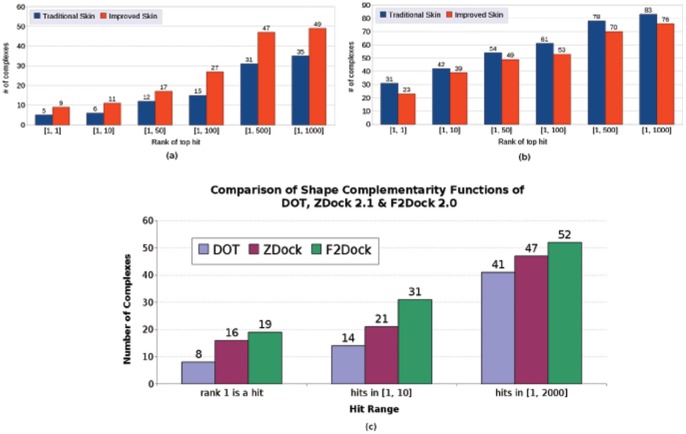
Effectiveness of the New Skin-Core Definition. (a–b) Comparison of the performance of F^2^ Dock 2.0’s shape complementarity function with traditional skin and the new floating skin approach, in terms of the number of complexes for which the top hit is within the ranges mentioned in the X-axis. (a) On the rigid-body unbound-(un)bound complexes from Zlab Benchmark 4.0. (b) On the rigid-body bound-bound complexes from Zlab Benchmark 4.0. (c) Comparison of the shape complementarity functions of DOT, ZDock 2.1 and F^2^ Dock 2.0 on the rigid-body bound-bound complexes from Zlab benchmark 2.0.

In Figure 3(a), we clearly notice the improvement offered by the floating skin approach over the traditional which validates our idea that a softer definition of skin is better for unbound docking. However, the traditional skin approach performs slightly better for the bound-bound (re-docking) test cases (Figure 3(b)). Figure 3(c) shows that as a result of the improved skin definition, F^2^ Dock 2.0’s shape complementarity function outperforms DOT and ZDock on the rigid complexes from Zlab benchmark 2.0 (bound-bound).

#### 3.10.2 Effects of various filters on quality of solutions

**Figure 4:**
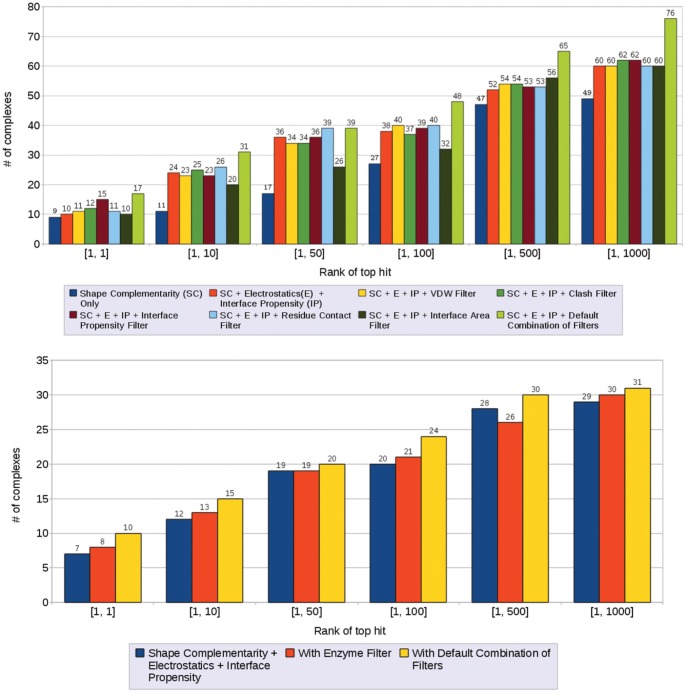
Analysis of the efficacy of the different filters and affinity terms used in F^2^ Dock 2.0. **(top)** Improvements in the rank of the top hit (of rigid-body test cases from Zlab benchmark 4.0) as various affinity functions and filters in F^2^ Dock 2.0 are activated one after another. **(bottom)** Improvements in the rank of the top hit for the Enzyme type of complexes from Zlab benchmark 4.0.

Figure 4(top) shows how the number of test cases (rigid-body test cases from Zlab benchmark 2.0 [36]) with at least one hit in top 1, top 10, top 50, top 100, top 500 and top 1000 changes as various affinity functions and filters in F^2^ Dock 2.0 are applied. The filters are applied to the top 2000 predictions after using the FFT based affinity terms and clustering. In this experiment, we have specified the complex type (A/AB, E and O) for each test case. Clearly, each of the filters (except interface area filter) individually improves the ranks of the top solution, and the best outcome is generated when the default combination of filters are used. For example, after the FFT based scoring, we get a hit at rank 1 for 10 complexes, but after filtering it improves to 17. Since the antibody and enzyme filters do not apply to all types of complexes, we compare their effect only on the particular type of complexes. For example, Figure 4(bottom) shows the effectiveness of the enzyme filter.

**Figure 5:**
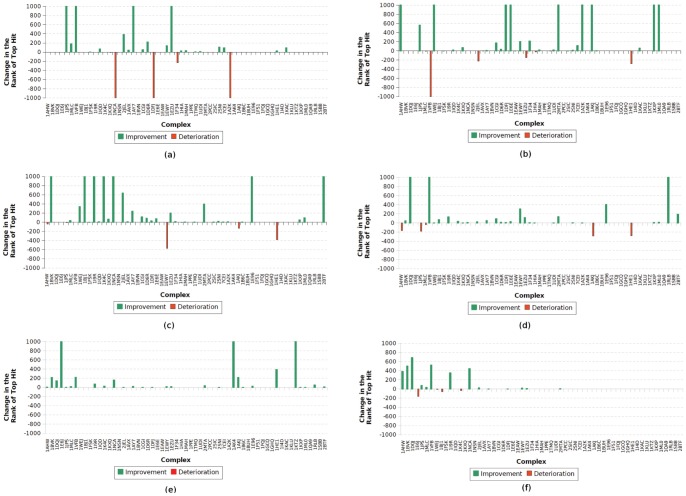
Changes in the rank of top hit as various options in F^2^ Dock 2.0 are activated one after another (on the rigid-body test cases from Zlab benchmark 2.0 [36]). (**a**) Lennard-Jones Filter (LJ), clash filter (CL) and proximity clustering (PC) are activated after shape complementarity (SC), (**b**) electrostatics & charge complementarity (EL) after SC+LJ+CL+PC, (**c**) interface propensity (IP) after SC+LJ+CL+PC+EL. (**d**) interface propensity filter (PF) after SC+LJ+CL+PC+EL+IP, (**e**) residue-residue contact filter (RC) after SC+LJ+CL+PC+EL+IP+PF, and (f) antibody contact filter (AF) or glycine filter (GF) after SC+LJ+CL+PC+EL+IP+PF+RC.

The series of plots in Figure 5 shows a detailed breakdown of the effect of different scores/filters for each complex separately. On the X-axis, we list the complexes and the Y-axis shows the change of the rank of the top hit. In the figures, an improvement is defined as producing the top hit at a better rank. We use the results of using just shape complementarity as the base case and analyze the relative improvements as more and more terms are added.

When we activate Lennard-Jones filter, clash filter and proximity clustering after shape complementarity we get hits for 4 new test cases, and the rank of the top hit improves for 15 more (see Figure 5(a)). However, we also lose hits in top 1000 for 3 test cases, and the rank of the top hit degrades for one test case. Overall, the application of these filters and clustering seem largely beneficial. The best results are obtained for enzyme-inhibitor/enzyme-substrate complexes, as for more than 50% of these complexes rank of the top hit improves.

When electrostatics is turned on we get hits in top 1000 for 9 test cases for which we did not have a single hit before, and for 14 other cases rank of the top hit improve (see Figure 5(b)). However, we lose hits 1 test case, and for 4 others rank of the top hit degrades.

The FFT-based interface propensity scoring is activated next which improves the rank of the top hit for 30 test cases (i.e., for around 50% of all cases) among which 7 cases did not have a single hit before (see Figure 5(c)). Among these 7 cases with new first hits 5 are antibody-antigen or antigen-bound antibody complexes, and none are enzyme-inhibitor or enzyme-substrate.

The interface propensity filter is turned on next. It improves the rank of the top hit for 25 complexes, and degrades for 5 (see Figure 5(d)). For 3 test cases we did not have a single hit in top 1000 before among which 2 are antibody-antigens.

The residue-residue contact filter which is activated next improves the rank of the top hit for 27 test cases, and degrades for none (see Figure 5(e)). The enzyme-inhibitor and enzyme-substrate complexes seem to have benefited the least from this filter.

Next we apply the antibody contact filer and the Glycine filter. The antibody contact filter improves the rank of the top hit for 9 antibody-antigen and antigen-bound antibody test cases, and degrades for 3, while the Glycine filter slightly improves the same for 4 enzyme-inhibitor/enzyme-substrate complexes (see Figure 5(f)).

More comparisons with respect to the RMSD of the best hit, the total number of hits, and the lowest RMSD are provided in Supplement S1.

#### 3.10.3 Effects of post-processing with GB-rerank

**Figure 6:**
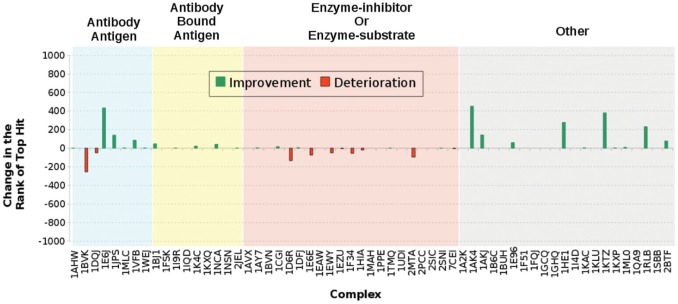
Effect of performing GBSA based reranking. The plot shows the change of the rank for the first hit. A positive change indicates that the reranker improves the result. For most complexes, specially for complexes where a knowledge-based based filter (Antibody or Enzyme) could not be applied, GB-rerank improves the rank of top hit compared to the results produced by F^2^ Dock 2.0 (for the rigid-body test cases from Zlab benchmark 2.0 [36]).

Figure 6 shows the impact of applying GB-rerank (after the initial docking phase) on the rigid-body test cases from Zlab benchmark 2.0 [36]. GB-rerank improves the ranks of the top hit for 9 antibody-antigen and antigen-bound antibody complexes, and 10 complexes of type “other” (see Figure 6).

The post-processor is least effective on enzyme-inhibitor/enzyme-substrate complexes since the enzyme filter has already improved the ranks quite well. On the other hand, for the ‘other’ complexes, GB-rerank produces the most significant improvements, since specific filters cannot be applied in these cases. Hence if the complex is known to be Enzyme, then GB-rerank should not be applied.

#### 3.10.4 Performance of F^2^ Dock 2.0 with and without user-specified complex type

**Figure 7:**
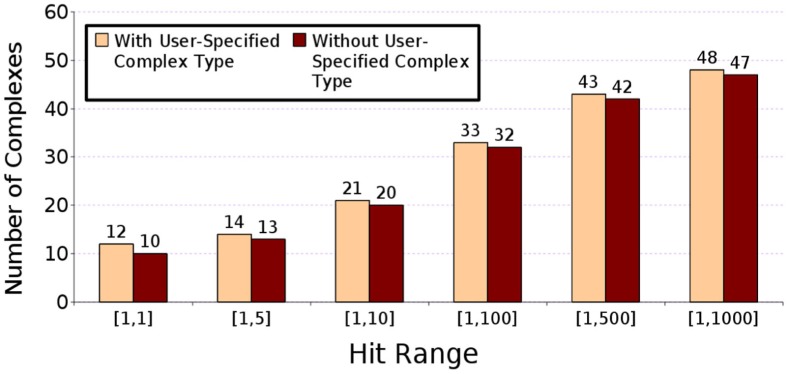
Performance of F^2^ Dock 2.0 with and without user-specified complex type. When complex type is not specified in the input, F^2^ Dock 2.0’s performance does not change significantly. In most cases, it can automatically detect the complex-type and apply the correct set of parameters. Tests are based on rigid body cases from Zlab’s Protein-protein docking Benchmark 2.0.

Figure 7 compares the performance of F^2^ Dock 2.0 with and without user-specified complex types on Zlab’s protein-protein docking benchmark 2.0. When no complex type is specified F^2^ Dock 2.0 tries to identify antibody-antigen complexes by locating the CDR loop regions of the antibody. Among the 17 such complexes in our experiments 16 are correctly identified by F^2^ Dock 2.0. It fails to identify 1KXQ which is an antibody-antigen complex from a Camelid (camels, llamas, etc.) [60]. Camelids produce functional antibodies that do not have light chains and CH1 domains, and so F^2^ Dock 2.0’s antibody detection system fails to identify such antibodies. Hence for 1KXQ the set of parameter values optimized for complexes of “other” type is applied, and the result is only slightly worse than what is obtained with the parameter set optimized for antibody-antigen complexes. F^2^ Dock 2.0 fails to select the correct parameter set for the following three enzyme-inhibitor/enzyme-substrate complexes among the 21 included in the experiments: 1AY7, 1UDI and 2MTA. While for 1UDI and 1AY7 F^2^ Dock 2.0 is still able to get a hit in the top 100 and top 500, respectively, it fails to get any hit in the top 1000 for 2MTA. For all other complexes the results remain the same except for 1WEJ for which we get slightly different results in the two set of experiments due to the non-determinism (arising from multiple concurrent threads) that exists in the proximity clustering phase.

### 3.11 Comparison with ZDock

In this section we compare the performance of F^2^ Dock 2.0 and ZDock 3.0.2 [21], [61] on the complexes from Zlab benchmark 4.0 [37]. We acquired the executable for ZDock 3.0.2 from their website and ran it following the steps specified in the accompanying instructions and used the PDB files downloaded from ZLab’s website without any modification. F^2^ Dock 2.0 used the same set of PDBs after performing the preprocessing we mentioned in Section 2.7. Note that ZDock 3.0.2 also applies their own preprocessing which is part of the mark_sur script provided with the executable. Both programs used 15° rotational sampling. F^2^ Dock 2.0 used user-specified complex types.

**Figure 8:**
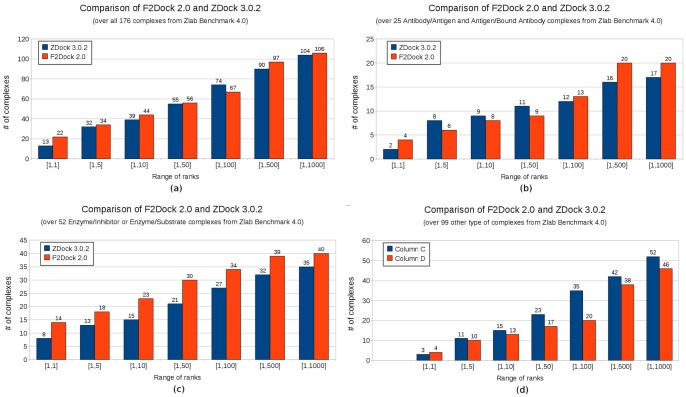
Comparison of ZDock 3.0.2 [21] and F^2^ Dock 2.0. (a) On all 176 complexes from Zlab Benchmark 4.0 [37], (b) On 25 antibody-antigen and antigen-bound antibody complexes, (c) On 52 enzyme-inhibitor and enzyme-substrate complexes, and (d) on the 99 other type of complexes.

In Figure 8, we show a summary of the performances in terms of the number of complexes where each protocol found at least one hit in different ranges (see the X-axis). Note that having a higher Y-axis value for any instance shows that the corresponding protocol is successful on complexes than the other. In Figure 8(a) we compare the performances over the entire Zlab benchmark 4.0 containing 176 complexes. We find that for each of the ranges except one, F^2^ Dock 2.0 performs better than ZDock 3.0.2. F^2^ Dock 2.0 is specially impressive since it gets a hit at rank 1 for 22 of the complexes (which is 1/8th of the dataset) as opposed to 13 found by ZDock 3.0.2. Overall both ZDock 3.0.2 and F^2^ Dock 2.0 finds at least one solution for about the same number of complexes, 104 and 106 respectively.

Figures 8(b)–(d) compares F^2^ Dock 2.0 and ZDock 3.0.2 using the same metrics but considers each type of complex separately. For antibodies there is not much to choose between the two protocols. For other types F^2^ Dock 2.0 is successful for a lower number of complexes, and is comparable only at relatively high ranks. However, for Enzymes, F^2^ Dock 2.0 completely outperforms ZDock 3.0.2 across the board.

Based on these results, we can clearly see that F^2^ Dock 2.0 produces much more reliable predictions for Enzymes, but there is not much difference for antibodies and other type of complexes. But Tables 1, 2, 3, and 4 show that even for antibodies and other types F^2^ Dock 2.0 provides significant contributions since the two protocols are often successful for different complexes and hence compliment each other. For example, among the antibodies, F^2^ Dock 2.0 finds a solution for 1QFW and 1I9R for which ZDock 3.0.2 does not find any solutions, on the other hand ZDock 3.0.2 finds a solution for 1NSN where F^2^ Dock 2.0 fails. Similarly among the other complexes, only F^2^ Dock 2.0 is successful for 1J2J, 2A5T, 2A9K, 2HQS, 3BP8, 1K5D, 1R6Q, 2Z0E, 3CPH and 1ATN. Hence, it is advisable to use both of these protocols specially for other type of complexes to increase the possibility of finding a correct solution.

**Table 1:** Comparison of the performance of F^2^ Dock 2.0 and ZDock 3.0.2 for each of the 25 antibody-antigen and antigen-bound antibody complexes from ZLab’s benchmark 4.0 in terms of the rank and RMSD of the top hit and the best hit.

Difficulty	Complex	Rank of		RMSD of		Rank of		Lowest	
		First Hit		First Hit		Lowest RMSD		RMSD	
		ZDock	F2Dock	ZDock	F2Dock	ZDock	F2Dock	ZDock	F2Dock
Easy	1AHW	354	**8**	4.5	**4.4**	1242	**457**	**0.9**	1.8
	1BJ1	**1**	63	**1.9**	2.3	**1**	63	**1.9**	2.3
	1BVK	**184**	205	**3.6**	4.9	358	**264**	**1.9**	4.1
	1DQJ	374	**74**	**4.0**	4.9	1787	**74**	**3.3**	3.6
	1E6J	**3**	126	**4.1**	5	181	**126**	**2.7**	5
	1FSK	1	1	**2.9**	3.2	**2**	3	1.8	**1.5**
	1I9R	–	**9**	–	**3.9**	–	**9**	–	**3.9**
	1IQD	18	**4**	4.3	**3**	68	**4**	**1.7**	3
	1JPS	1266	**186**	**2.1**	2.7	1266	**186**	**2.1**	2.7
	1K4C	583	**105**	**2.9**	4.4	583	**165**	2.9	**2.2**
	1KXQ	2	**1**	**1.2**	1.6	2	**1**	**1.2**	1.6
	1MLC	57	**11**	**2.0**	3.8	**57**	114	2.0	**1.3**
	1NCA	**11**	168	**1.7**	3.7	**11**	168	**1.7**	3.7
	1NSN	**1267**	–	**1.6**	–	**1267**	–	**1.6**	–
	1QFW	–	**80**	–	**1.9**	–	**80**	–	**1.9**
	1VFB	250	**191**	**3.1**	4.8	560	**434**	**2.9**	3.4
	1WEJ	9	**5**	**1.5**	3.2	9	**5**	**1.5**	3.2
	2FD6	**3**	62	5.0	**4.4**	282	**62**	**3.3**	4.4
	2I25	**2**	122	**3.0**	3.9	**40**	242	**1.7**	2.6
	2JEL	4	**1**	3.5	**3.3**	753	**1**	**2.6**	3.3
	2VIS	–	–	–	–	–	–	–	–
	9QFW	2	**1**	4.0	**3.9**	48	**3**	**1.9**	2.9
Medium	1BGX	–	–	–	–	–	–	–	–
Hard	1E4K	–	–	–	–	–	–	–	–
	2HMI	–	–	–	–	–	–	–	–

Boldfaced entries indicate better performance on the particular metric for the complex. Empty entries indicate that no hits were found for that complex by the corresponding protocol.

**Table 2:** Comparison of the performance of F^2^ Dock 2.0 and ZDock 3.0.2 for each of the 52 enzyme-inhibitor and enzyme-substrate complexes from ZLab’s benchmark 4.0 in terms of the rank and RMSD of the top hit and the best hit.

Difficulty	Complex	Rank of		RMSD of		Rank of		Lowest	
		First Hit		First Hit		Lowest RMSD		RMSD	
		ZDock	F2Dock	ZDock	F2Dock	ZDock	F2Dock	ZDock	F2Dock
Easy	1AVX	25	**1**	**3.5**	4.5	194	**4**	**1.5**	2
	1AY7	577	**2**	**2.5**	4	577	**6**	**2.5**	2.5
	1BVN	3	**1**	**1.2**	3.2	3	**2**	**1.2**	3
	1CGI	**10**	76	4.0	**3.4**	**173**	199	**2.6**	3.3
	1CLV	3	**1**	**2.3**	2.5	**21**	350	2.3	**2.1**
	1D6R	–	**59**	–	**4.6**	–	**249**	–	**4.3**
	1DFJ	**1**	9	**4.1**	4.4	**2**	9	**3.2**	4.4
	1E6E	**5**	20	**3.2**	4.7	**10**	20	**1.5**	3.9
	1EAW	68	**1**	3.4	**1**	579	**1**	1.7	**1**
	1EWY	53	**14**	4.2	**3.2**	231	**14**	3.6	**3.2**
	1EZU	841	**170**	4.9	**4.5**	**841**	1554	4.9	**3.8**
	1F34	62	**2**	**3.4**	4.3	925	**3**	**2.4**	3.7
	1FLE	31	**3**	5.0	**3.7**	1102	**192**	3.4	**3**
	1GL1	**73**	326	**2.6**	3.8	**73**	881	2.6	**2.3**
	1GXD	**1173**	–	**4.9**	–	**1173**	–	**4.9**	–
	1HIA	–	**18**	–	**3.4**	–	**258**	–	**2.2**
	1JTG	**1**	7	**2.6**	4.6	1	**1173**	**2.6**	3.4
	1MAH	1	1	3.1	**2.7**	4	4	**1.4**	1.9
	1N8O	**7**	11	**3.4**	4.8	**20**	1330	**0.6**	4
	1OC0	**1590**	–	4.8	–	**1590**	–	**4.8**	–
	1OPH	**1694**	–	**3.9**	–	**1694**	–	**3.9**	–
	1OYV	15	**7**	4.9	**3.6**	153	**105**	3.3	**2.9**
	1PPE	1	1	2.9	**2.3**	3	3	**1.1**	1.3
	1R0R	138	**39**	**2.2**	4.3	1298	**1164**	2.0	**1.4**
	1TMQ	16	**1**	**3.6**	4.8	885	**515**	3.0	**2.4**
	1UDI	24	**1**	3.5	**3.1**	**24**	229	3.5	**2.5**
	1YVB	**1**	–	**2.4**	–	**18**	–	**2.2**	–
	2ABZ	–	**5**	–	**2.8**	–	**5**	–	**2.7**
	2B42	3	**1**	4.2	**3.9**	**6**	12	**0.6**	2.2
	2J0T	–	**19**	–	**2.8**	–	**21**	–	**2.6**
	2MTA	**76**	90	4.4	**4.3**	716	**100**	**0.7**	3.7
	2O8V	**29**	654	5.0	**3.7**	852	**654**	4.0	**3.7**
	2OUL	1	1	**1.7**	4.9	**1**	329	**1.7**	3
	2PCC	496	**10**	**2.6**	4.3	496	**10**	**2.6**	4.3
	2SIC	5	**1**	1.1	**1.1**	5	**1**	1.1	**1.1**
	2SNI	177	**1**	**3.8**	4.7	**299**	403	2.8	**1.3**
	2UUY	693	**7**	4.4	**4.1**	1946	**44**	3.1	**3**
	3SGQ	428	**110**	4.0	**2.6**	**576**	624	**1.0**	2
	4CPA	1	1	**4.4**	4.8	465	**202**	2.5	**2.4**
	7CEI	1	1	4.4	**4.1**	88	**2**	**0.8**	1.4
	BOYV	–	**220**	–	**3.6**	–	**220**	–	**3.6**
Medium	1ACB	126	**22**	4.4	**3.2**	393	**49**	**2.6**	2.6
	1IJK	**81**	88	**3.0**	4.8	1317	**142**	**2.0**	3.4
	1JIW	–	–	–	–	–	–	–	–
	1KKL	–	–	–	–	–	–	–	–
	1M10	–	–	–	–	–	–	–	–
	1NW9	–	**321**	–	**4.9**	–	**321**	–	**2.3**
Hard	1F6M	–	–	–	–	–	–	–	–
	1FQ1	–	–	–	–	–	–	–	–
	1PXV	–	–	–	–	–	–	–	–
	1ZLI	–	–	–	–	–	–	–	–
	2O3B	–	–	–	–	–	–	–	–

**Table 3:** Comparison of the performance of F^2^ Dock 2.0 and ZDock 3.0.2 for each of the 99 other type of complexes from ZLab’s benchmark 4.0 in terms of the rank and RMSD of the top hit and the best hit.

Difficulty	Complex	Rank of		RMSD of		Rank of		Lowest	
		First Hit		First Hit		Lowest RMSD		RMSD	
		ZDock	F2Dock	ZDock	F2Dock	ZDock	F2Dock	ZDock	F2Dock
Easy	1A2K	1348	**44**	4.3	**2.4**	1894	**44**	3.2	**2.4**
	1AK4	1090	**964**	**3.5**	4.3	1090	**964**	**3.5**	4.3
	1AKJ	546	**39**	**2.9**	3.4	1632	**39**	**1.7**	3.4
	1AZS	**42**	–	**2.9**	–	**61**	–	**2.0**	–
	1B6C	**1**	3	**2.9**	4	**1**	3	**2.9**	4
	1BUH	**30**	431	**3.6**	4.8	1961	**431**	**3.0**	4.8
	1E96	1171	**278**	**3.8**	5	1171	**314**	**3.8**	4.2
	1EFN	–	–	–	–	–	–	–	–
	1F51	**589**	–	**4.6**	–	**589**	–	**4.6**	–
	1FC2	–	**1190**	–	**5**	–	**1570**	-	**4.1**
	1FCC	–	–	–	–	–	–	–	–
	1FFW	**73**	325	**4.5**	4.7	1349	**1291**	4.0	**3**
	1FQJ	–	–	–	–	–	–	–	–
	1GCQ	**1105**	–	**1.4**	–	**1105**	–	**1.4**	–
	1GHQ	–	–	–	–	–	–	–	–
	1GLA	**1708**	–	**3.9**	–	**1708**	–	**3.9**	–
	1GPW	3	**1**	**3.6**	3.7	134	**5**	**2.1**	2.6
	1H9D	**1006**	–	**4.5**	–	**1006**	–	**4.5**	–
	1HCF	**175**	1225	**4.0**	4.7	**225**	1225	**1.9**	4.7
	1HE1	1141	**574**	**4.7**	4.9	1141	**574**	**4.7**	4.9
	1I4D	**571**	–	**4.2**	–	**571**	–	**4.2**	–
	1J2J	–	**182**	–	**4.6**	–	**182**	–	**4.6**
	1JWH	**7**	–	**3.6**	–	**78**	–	**1.9**	–
	1K74	**2**	3	**1.2**	3.8	**2**	7	**1.2**	2.6
	1KAC	592	**8**	4.5	**4.4**	1527	**99**	**1.9**	4.1
	1KLU	**1957**	–	**3.4**	–	**1957**	–	**3.4**	–
	1KTZ	535	**98**	**2.8**	3.9	535	**166**	**2.8**	2.9
	1KXP	**1**	7	**1.6**	3.5	**1**	260	**1.6**	2.6
	1ML0	4	**2**	**3.1**	4.3	**8**	123	**3.1**	3.2
	1OFU	**84**	–	**4.5**	–	**347**	–	**3.1**	–
	1PVH	**748**	–	**4.5**	–	**1192**	–	**1.5**	–
	1QA9	–	–	–	–	–	–	–	–
	1RLB	**3**	555	**4.6**	5	**232**	555	**3.4**	5
	1RV6	2	2	**1.3**	4	**2**	694	**1.3**	2.2
	1S1Q	**756**	–	**1.9**	–	**1243**	–	**1.4**	–
	1SBB	–	–	–	–	–	–	–	–
	1T6B	**58**	525	**3.6**	4	1510	**752**	2.8	**2.7**
	1US7	**74**	–	**1.1**	–	**74**	–	**1.1**	–
	1WDW	2	**1**	**1.2**	2.5	2	**1**	**1.2**	2.5
	1XD3	8	**1**	**4.0**	4.2	**86**	1298	**2.6**	3.9
	1XU1	**912**	–	**5.0**	–	**912**	–	**5.0**	–
	1Z0K	**8**	307	**3.3**	3.3	**8**	307	**3.3**	3.3
	1Z5Y	**20**	–	**3.4**	–	423	–	2.5	–
	1ZHH	–	–	–	–	–	–	–	–
	1ZHI	**65**	202	4.4	**4**	324	**202**	**2.1**	4
	2A5T	–	**268**	–	**3.6**	–	**618**	–	**2.9**
	2A9K	–	**558**	–	**3.4**	–	**558**	–	**3.4**
	2AJF	**475**	–	**3.6**	–	**475**	–	**3.6**	–
	2AYO	**37**	1108	3.3	**2**	**138**	1108	2.5	**2**
	2B4J	–	–	–	–	–	–	–	–
	2BTF	**53**	95	4.7	**4.5**	**148**	377	3.8	**3.4**
	2FJU	261	**228**	**3.2**	4.2	**261**	333	**3.2**	3.5

**Table 4:** Comparison of the performance of F^2^ Dock 2.0 and ZDock 3.0.2 for each of the 99 other type of complexes from ZLab’s benchmark 4.0 in terms of the rank and RMSD of the top hit and the best hit.

Difficulty	Complex	Rank of		RMSD of		Rank of		Lowest	
		First Hit		First Hit		Lowest RMSD		RMSD	
		ZDock	F2Dock	ZDock	F2Dock	ZDock	F2Dock	ZDock	F2Dock
Easy	2G77	15	**8**	**1.5**	3.7	**15**	917	1.5	**1.3**
Cont	2HLE	31	**4**	4.1	**3.8**	31	**4**	4.1	**3.8**
	2HQS	–	**27**	–	**4.1**	–	**125**	–	**2.7**
	2OOB	–	–	–	–	–	–	–	–
	2OOR	766	**16**	4.4	**4**	766	**63**	4.4	**2**
	2VDB	**5**	–	**1.2**	–	**5**	–	**1.2**	–
	3BP8	–	**474**	–	**5**	–	**699**	–	**3.3**
	3D5S	71	**1**	**3.1**	3.2	609	**4**	**2.5**	2.7
Medium	1GP2	**61**	193	4.4	**4**	**107**	193	**2.8**	4
	1GRN	1299	**401**	**4.3**	4.8	1299	**401**	**4.3**	4.8
	1HE8	–	–	–	–	–	–	–	–
	1I2M	**267**	545	**2.2**	2.6	**267**	545	**2.2**	2.6
	1IB1	–	–	–	–	–	–	–	–
	1K5D	–	**521**	–	**4.3**	–	**521**	–	**4.3**
	1LFD	**85**	990	4.6	**4.6**	**466**	1235	4.5	**4.1**
	1MQ8	**1455**	–	**3.2**	–	**1455**	–	**3.2**	–
	1N2C	–	–	–	–	–	–	–	–
	1R6Q	–	**180**	–	**3.7**	–	3**11**	–	**3.5**
	1SYX	211	**2**	4.8	**4.7**	211	**11**	4.8	**3**
	1WQ1	**81**	–	**4.0**	–	**81**	–	**4.0**	–
	1XQS	**19**	61	**3.8**	4.2	**45**	833	**2.6**	3.7
	1ZM4	**6**	–	**4.1**	–	**631**	–	**2.7**	–
	2CFH	**1**	119	3.8	**2.6**	**2**	119	**1.7**	2.6
	2H7V	**1112**	–	**4.6**	–	**1112**	–	**4.6**	–
	2HRK	**3**	–	**3.7**	–	**3**	–	**3.7**	–
	2J7P	–	–	–	–	–	–	–	–
	2NZ8	**64**	–	**4.5**	–	**64**	–	**4.5**	–
	2OZA	–	–	–	–	–	–	–	–
	2Z0E	–	**169**	–	**3.9**	–	**169**	–	**3.9**
	3CPH	–	**250**	–	**4.3**	–	**250**	–	**4.3**
Hard	1ATN	–	**1307**	–	**2.7**	–	**1307**	–	**2.7**
	1BKD	–	–	–	–	–	–	–	–
	1DE4	**84**	–	**4.7**	–	**84**	–	**4.7**	–
	1EER	–	–	–	–	–	–	–	–
	1FAK	–	–	–	–	–	–	–	–
	1H1V	–	–	–	–	–	–	–	–
	1IBR	–	–	–	–	–	–	–	–
	1IRA	–	–	–	–	–	–	–	–
	1JK9	510	**422**	4.2	**2.5**	790	**422**	4.1	**2.5**
	1JMO	–	–	–	–	–	–	–	–
	1JZD	**44**	144	4.6	**4**	**44**	144	4.6	**4**
	1R8S	–	–	–	–	–	–	–	–
	1Y64	–	–	–	–	–	–	–	–
	2C0L	–	–	–	–	–	–	–	–
	2I9B	–	–	–	–	–	–	–	–
	2IDO	**130**	156	**3.6**	4.5	**154**	156	**3.5**	4.5
	2OT3	**121**	–	**4.6**	–	**327**	–	**4.5**	–

**Figure 9:**
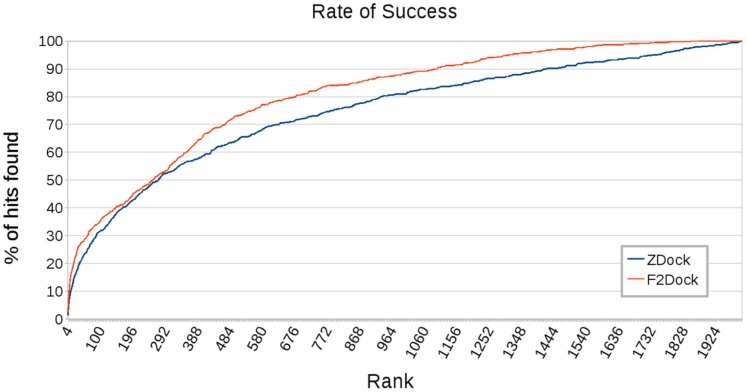
Comparison of the rate of success of F^2^ Dock 2.0 and ZDock 3.0.2. On the 176 complexes from ZLab’s benchmark 4.0. Rate of success is defined as the percentage of the hits found within the top 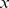 ranks, where 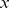 is the corresponding value of the X-axis. Clearly F^2^ Dock 2.0 has a better ratio.

Next, we compare the rate of success of the two protocols. Let us assume that the total number of hits (counting multiple hits found for a complex) found within a range 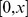 across all the complexes be 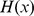. Now we define the rate of success as 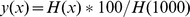 which measures how quickly a protocol finds its hits. A protocol with a higher ratio has higher true positive rate near the top of the list. If we plot this function, we expect to see a curve which rises sharply and then gradually flattens and converges to 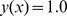. In Figure 9, we see that F^2^ Dock 2.0 has consistently better success rate than ZDock 3.0.2.

A closer look at Tables 1, 2, 3, and 4 shows that the RMSDs of the predictions by F^2^ Dock 2.0 is poorer than ZDock 3.0.2 in more occasions than it is better. This is due to our softer skin approach which rewards docking poses which have slightly larger gap between them, and our stringent clash and VDW filters which discard ligand poses which comes too close. This is beneficial for unbound complexes with larger conformational change, but prevents ligands of rigid (easy cases in the benchmark) from getting as close as they could be placed. The result clearly shows that ZDock 3.0.2 gets better RMSDs for rigid cases, and F^2^ Dock 2.0 is better for non-rigid cases. At this point, it should be mentioned that F^2^ Dock 2.0 is designed solely as a initial stage docking tool, which can quickly perform exhaustive search and return good leads at high ranks. Hence the poses it finds are generally acceptable or medium quality as defined in the criteria used in the CAPRI [62] challenge (tables summarizing F^2^ Dock 2.0’s performance using the CAPRI criteria can be found in Supplement S1). Local refinements (rigid body or flexible) can then be performed on a small number of top solutions to further improve their RMSDs and minimize the energies. There are a host of such tools available including ROSETTA [10], Amber [59], FireDock [63] etc.

We conclude this section with the observation that F^2^ Dock 2.0 shows better overall performance, with significant improvement for Enzymes. For other type of complexes the performance is comparable and sometimes complementary.

### 3.12 Running Times

**Figure 10:**
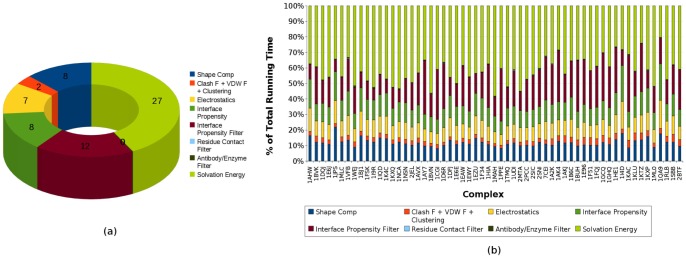
Running time of F^2^ Dock 2.0 and its components. (**a**) Average running time of each affinity function and filter of F^2^ Dock 2.0. GB-rerank consumes a major portion of the time (42%), the FFT phase takes about 30% time and the rest is taken by the filters and clustering. The labels in the figure are actual time in minutes. (**b**) Running times of F^2^ Dock 2.0 on the rigid-body test cases from Zlab benchmark 2.0 [36] showing percentage of running time due to each affinity function and filter of F^2^ Dock 2.0 for each complex.

To evaluate the average running times and the relative consumption by each scoring term/filter we performed a set of experiments run on a 3 GHz 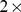
dual-core (i.e., 4 cores) AMD Opteron 2222 processor with 4 GB RAM. On average, the FFT phase took around 23 minutes or 35% of the total running time, the interface propensity filter took 20%, GB-rerank accounted for around 42%, and the remaining 3% is spent on the other filters. GB-rerank and interface propensity filter take longer to compute than other filters, since the computation is based on surface quadrature points, whose number is a constant multiple of the number of atoms. Figure 10 shows how the different components of F^2^ Dock 2.0 and GB-rerank contribute to the total running time of the docking and reranking process on the rigid-body test cases from Zlab benchmark 2.0 [36]. Overall, about 30% time is taken up by the FFT based affinity functions, 30% is taken up the the filters (mostly the interface propensity filter), and around 40% by the GB-rerank.

F^2^ Dock 2.0 leverages from the embarrassingly parallel nature of the computation using multithreaded computations on multi-core machines. Note that each of the 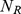 FFT computations are independent of each other and can be run in parallel. Scores for each of filter terms for each of the poses in 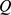 can also be computed in parallel. Specifically, given 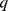 cores and 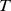 tasks the simplest strategy is to distribute 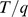 tasks to each cores. But this approach often leads to unbalanced exploitation of the cores if the tasks given to different cores take different amount of time to complete. For example, the running times of the filters are proportional to the size of the interface which varies between different poses. So our technique initially sends only one task to each core and maintains a queue of remaining tasks, and then whenever a core is done with its task, it gets another one from the queue. This scheduling ensures that every core is exploited equally and hence the overall completion time is quicker.

### Conclusions

We have developed an enhanced version (F^2^ Dock 2.0 ) of our protein-protein docking program F^2^ Dock 2.0 with improved scoring functions, complete with dynamic clustering and filtering and generalized Born based solvation energetic reranking. The on-the-fly FFT-based scoring function is a weighted combination of shape-complementarity, Coulombic electrostatics complementarity, and interface propensity terms. The on-the-fly docking also includes filters based on Lennard-Jones potential, steric clashes, residue-residue contact statistics and an extremely fast approximation of solvation energy using a newly developed fast multipole type implementation with octree data structures. Our implementation results and numerous tests show that each of these terms and filters significantly improves the accuracy of docking predictions. Our use of highly efficient data structures including the dynamic packing grids for near constant time neighborhood search and near-far distance clustering using octrees, significantly speed up the computations for each of the ‘on-the-fly’ scoring and filtering terms. GB-rerank ’s solvation energy based post-processing suite is also optimized using these efficient data structures with the best tradeoffs of docking accuracy vs. speed. The entire software is highly parallel and can be run efficiently on multicores and clusters of multicores (e.g., many modern supercomputers). We have also developed a GUI based interface (TexMol) for easily preparing and running a docking process and interactively visualize, compare different solutions along with several relevant statistics including interface area, residue contacts, binding energy etc.

### 4.13 Future Work

Currently F^2^ Dock 2.0 addresses flexibility by simply performing a ‘soft’ complementarity with the goal of identifying a near-native solution at a higher rank. The solutions can be optimized further by side chain refinement near the binding site as well as applying small rigid-body perturbations which moves all atoms of the ligand. There are several software including AMBER [59], ROSETTA [10], SCRWL [64] etc. which can be used to achieve this objective. We are currently working on an algorithm for dead-end elimination with better complexities compared to SCRWL. The automatic assignment of all the docking parameters remains a very active area of research for us; we are currently pursuing a semi-supervised computational learning algorithm over the space of different protein families which will further improve the performance of F^2^ Dock 2.0 and GB-rerank. We are exploring ways to further improve the speed of F^2^ Dock 2.0 specially using GPU level parallelism. We are also actively working on extending F^2^ Dock 2.0 to Protein-RNA and RNA-RNA docking. Finally, a web service supporting similar features as TexMol is under construction.

## Supporting Information

Supplement S1 **Supplemental materials.**
(PDF)
