# Supplementary material for: Protein-Protein Docking with F2Dock 2.0 and GB-Rerank
Source: PLoS One. 2013 Mar 6;8(3):e51307. doi: 10.1371/journal.pone.0051307 (PMC3590208; doi:10.1371/journal.pone.0051307)
Supplement: Supplement S1 — Supplemental materials. (PDF) [file pone.0051307.s001.pdf]

Protein-Protein Docking with F^2Dock 2.0 and GB-rerank

Supplement

Rezaul Chowdhury[1], Muhibur Rasheed[1], Donald Keidel[2], Maysam Moussalem[1], Arthur Olson[2], Michel Sanner[2], Chandrajit Bajaj[2,*]

1 Department of Computer Science, University of Texas at Austin, 1 University Station C0500, Austin, Texas 78712, USA
2 The SCRIPPS Research Institute, Torrey Pines Rd N, La Jolla, CA 92037, USA
∗ E-mail: bajaj@cs.utexas.edu

Contents

1 Details on Affinity Function Computations 2
- 1.1 Shape Complementarity . . . 2
  - 1.1.1 Skin-core Definition and Weighting . . . 2
  - 1.1.2 FFT based formulation . . . 4
- 1.2 Electrostatics (E). . . . 5
  - 1.2.1 FFT based formulation . . . 5
- 1.3 Interface Propensity (IP) and Hydrophobicity (HP). . . . 6
  - 1.3.1 FFT based formulation . . . 6

2 Details on Filters 8
- 2.1 Lennard-Jones Filter. . . . 8
- 2.2 Interface Area Filter. . . . 9
- 2.3 Interface Propensity Filter. . . . 9
- 2.4 Clash Filter. . . . 10
- 2.5 Residue-Residue Contact Filter. . . . 10
- 2.6 Glycine Filter. . . . 11
- 2.7 Antibody-Antigen Contact Filter. . . . 11
- 2.8 Overall Cost of Filtering. . . . 11

3 Solvation Energy Based Reranking 12
- 3.1 Approximating E_{pol} (Polarization Energy). . . . 12
- 3.2 Approximating $E_{\text{vdw(s-s)}}$ (Dispersion Energy). . . . 13
- 3.3 Approximating E_{cav} (Cavity Forming Energy). . . . 13
- 3.4 Overall Cost of Reranking. . . . 14

4 Additional Results on the Effects of Various Affinity Functions and Filters 15

5 Additional Results Comparing ZDock 3.0.2 and F^2Dock 2.0 25

1 Details on Affinity Function Computations

This section adds detail on the FFT based implementation and parameter selection of three affinity functions: shape complementarity, electrostatics and interface propensity (or hydrophobicity).

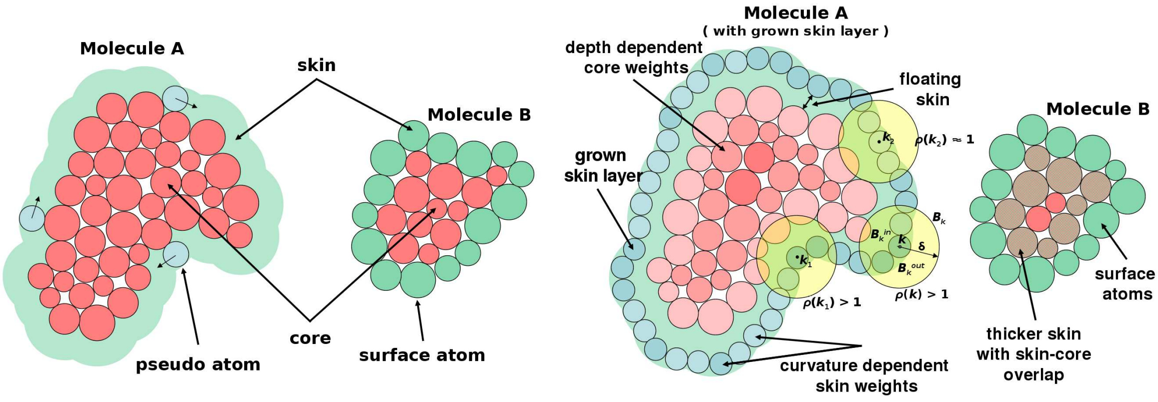

Figure S1: **Traditional vs improved double-skin definitions.** (Left) Traditional *double skin-layer* approach for shape complementarity, (Right) Curvature-based weighting of skin atoms and depth dependent weighting of core atoms of molecule A, and depth dependent weighting of the atoms of B

1.1 Shape Complementarity

First we present the new skin-core definitions in detail.

1.1.1 Skin-core Definition and Weighting

- Floating Receptor (i.e., Stationary Molecule A) Skin: Unlike the traditional approach [1] the receptor skin layer does not touch the receptor van der Waals surface (i.e., there is a gap between the skin and the core regions), and also the radius of those skin atoms differ from that in the traditional approach. The plots in Figure S2 justify this skin-core gap which, for each of 60 rigid-body test cases chosen from Zlab Benchmark Suite 2.0 [2], plots average distance and standard deviation of ligand atom centers from the receptor van der Waals surface when the ligand is bound to the receptor. We have considered only those ligand atoms that lie within 3Å from the receptor surface in bound condition. For the bound-bound case (when both molecules are crystallized together in the complex form) the average distance lies between 1.6Å and 2.0Å, while for unbound-unbound case (when the two molecules are crystallized separately) it lies between 1.4Å and 2.0Å most of the time. The standard deviation lies between 0.55Å and 0.75Å for the bound-bound case, and between 0.6Å and 1Å for the unbound-unbound case. In our experiments pseudo skin atoms of radius 1.1Å with their centers placed at a distance of 1.7Å from the receptor vdW surface (so there is a gap of 0.6Å) performed much better than the traditional approach where pseudo atoms of radius 1.4Å are placed touching the receptor vdW surface.

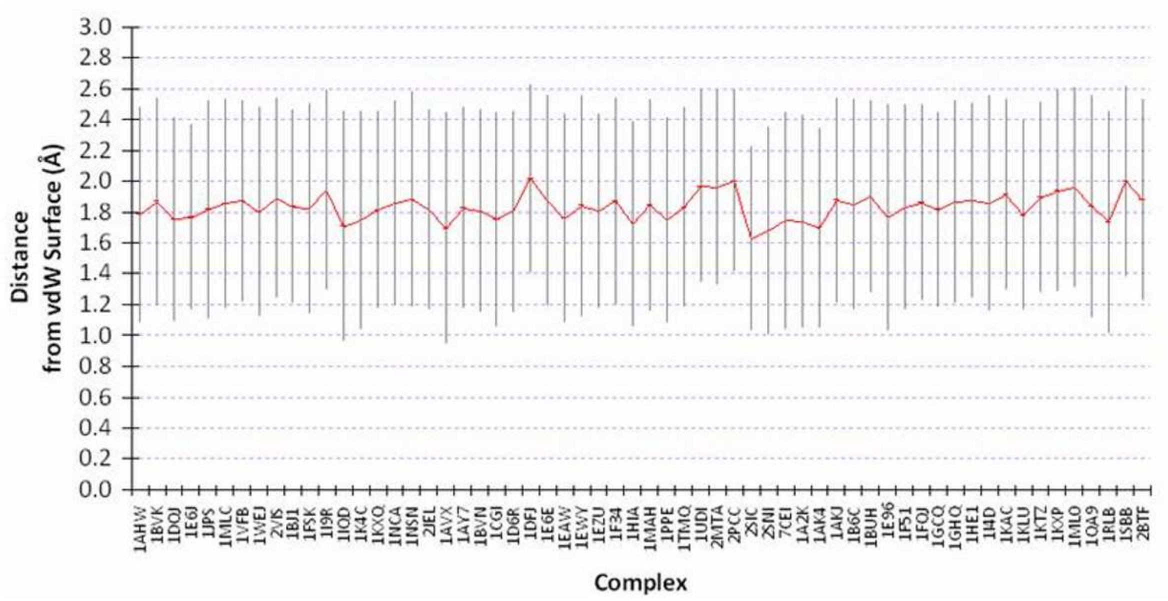

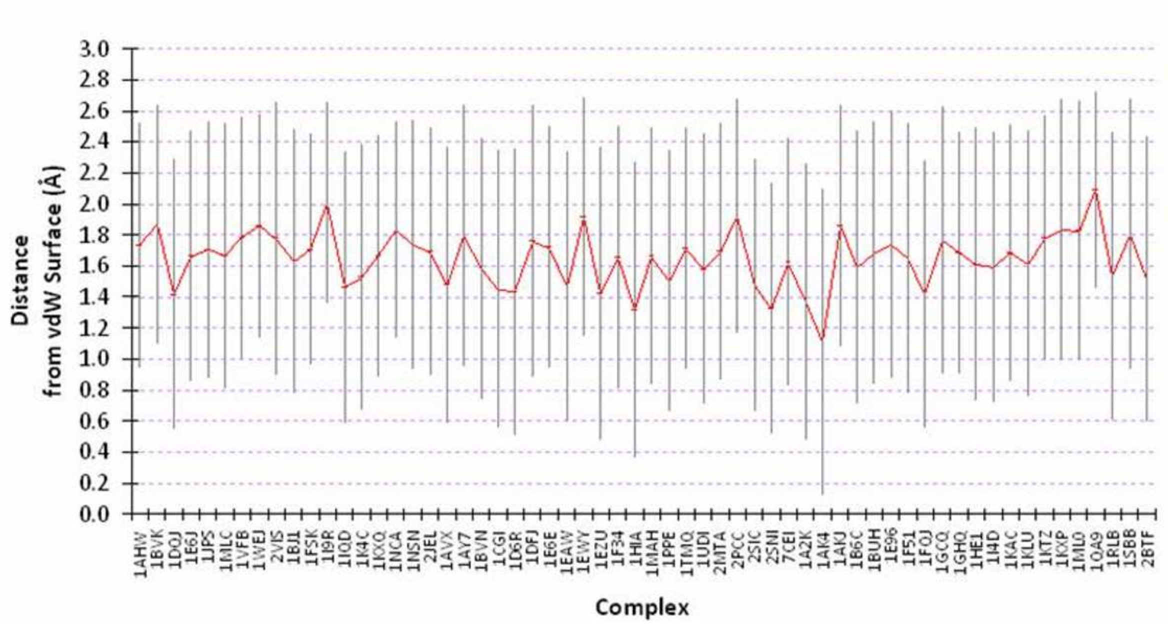

Figure S2: **Interface gap for bound and unbound complexes in benchmark 4.0**. Plots show average distance (in red) and standard deviation of ligand atom centers from the receptor van der Waals surface when the ligand is bound to the receptor. Only the ligand atoms that lie within 3Å from the receptor surface in bound condition are considered. The plots are for the rigid-body test cases from Zlab Benchmark Suite 2.0 [2]. Top: Bound-bound. Bottom: Unbound-unbound

- Curvature dependent Weighting of Receptor Skin: The weights assigned to receptor skin atoms are computed based on the curvature of the skin around that atom (as opposed to

the constant weights assigned to skin atoms in the traditional approach [1]). Such weighting encourages convex-concave and concave-convex interfaces as opposed to large flat interfaces.

We know that the centers of molecule A's skin atoms are placed at a constant distance d from the receptor vdW surface S. Let us enlarge S outward by distance d so that it passes through the skin atom centers, and let V be the volume enclosed by the enlarged surface. Let B_k be a ball of radius δ at the center of skin atom k, and $B_k^{in} = B \cap V$ and $B_k^{out} = V \setminus B_k^{in}$. We define:
$\rho(k) = \max\left\{ \frac{|B_k^{in}|}{|B_k^{out}|}, \frac{|B_k^{out}|}{|B_k^{in}|} \right\}$.

Then the weight assigned to skin atom k of molecule A is $c_{A,k}^{SC} = \rho(k)$.

We approximate $\rho(k)$ from the 3D grid embedding of molecule A along with its skin region, and $\delta = 3.6$ produced good results in our experiments.

- Depth dependent Weighting of Receptor Core: The core atoms of molecule A are assigned weights using an increasing function of depth (distance of the atom center from the surface of A). Such weightings discourage deeper core-core overlaps more compared to shallower ones by making it difficult to compensate for deep core-core overlaps with wide skin-skin overlaps.

  The core atom weights are computed based on *atomic layers*. Any atom which contributes to the solvent excluded surface (SES) is considered an exposed atom. The exposed atoms of a molecule lie in layer 1. When atoms in layer 1 are removed, the new exposed atoms make layer 2. In general, when atoms in layers 1 to $l \geq 1$ are removed, the exposed atoms among the remaining ones make layer $l+1$. The atomic layer of core atom k of A is denoted by $L_A(k)$, and the weight assigned to that atom is $c_{A,k}^{SC} = i \cdot \alpha^{L_A(k)}$, where $\alpha \geq 1$ is a constant. A value of $\alpha = 1.1$ worked quite well in our experiments.

  Hence overall, the weight assigned to atom/pseudo-atom k of molecule A:

$$c_{A,k}^{SC} = \begin{cases} \rho(k) & \text{if atom } k \in skin(A), \\ i \cdot \alpha^{L_A(k)} & \text{otherwise.} \end{cases}$$

- Thicker Skin of the Ligand (i.e., Moving Molecule B) with Skin-Core Overlap: Since in the traditional approach [1] the ligand skin is defined using its surface atoms, the skin thickness varies and can be too thin in some areas. It turns out that a double layer ligand skin with skin-core overlap performs better than a single skin layer disjoint from the core. The weights assigned to the atoms of B are as follows.

$$c_{B,k}^{SC} = \begin{cases} 1 & \text{if } L_B(k) = 1, \\ 1+i & \text{if } L_B(k) = 2, \\ i & \text{otherwise.} \end{cases}$$

1.1.2 FFT based formulation

Now suppose the user-specified relative weights for various types of overlaps are as follows:

w_{ss} = reward for (unit) skin-skin overlap,

w_{cc} = penalty for (unit) core-core overlap, and

w_{sc} = reward/penalty for (unit) skin-core overlap.

Let A' denote molecule A with its grown skin layer. If the weight assigned to an atom/pseudo-atom k (of molecule $P \in \{A', B\}$) is $c_k^{SC} = c_{k,Re}^{SC} + i \cdot c_{k,Im}^{SC}$, then the affinity function for shape complementarity is:

$$f_P^{SC}(\mathbf{x}) = \sum_{k \in P} \left(\sqrt{w_{ss}} \cdot c_{k,Re}^{SC} + i \cdot \sqrt{w_{cc}} \cdot c_{k,Im}^{SC}\right) \cdot g_{P,k}^{SC}(\mathbf{x}),$$

where, $g_{P,k}^{SC}(\mathbf{x}) = e^{-\beta\left(\frac{(\mathbf{x}-\mathbf{c}_k)^2}{r_k^2}-1\right)}$ with blobbiness $\beta = 2.3$.

The overall shape complementarity score for translation $\mathbf{t}$ and rotation $\mathbf{r}$ is:

$$H_{A,B}^{SC}(\mathbf{t},\mathbf{r}) = Re\left(F_{A,B}^{SC}(\mathbf{t},\mathbf{r})\right) + \frac{w_{sc}}{\sqrt{w_{ss} \cdot w_{cc}}} \cdot Im\left(F_{A,B}^{SC}(\mathbf{t},\mathbf{r})\right),$$

where, $F_{A,B}^{SC}(\mathbf{t},\mathbf{r}) = \int_{\mathbf{x}} f_A^{SC}(\mathbf{x}) T_{\mathbf{t}}\left(\Delta_{\mathbf{r}}\left(f_B^{SC}(\mathbf{x})\right)\right)$ with T and Δ being the translation and the rotation operator, respectively.

1.2 Electrostatics (E).

In [3] Gabb et. al. described a simplified model for electrostatics which allows efficient FFT-based computation of the term during docking search. We used this simple electrostatics model in the original version of F^2Dock 2.0 [1]. The first protein's electric potential is computed and matched against the charges in the other. Charge assignments are made using PDB2PQR [4]).

Two affinity functions f_A^E and f_B^E are defined for molecule A and B, respectively.

$$f_A^E(\mathbf{x}) = \sum_{k \in A} \frac{q_k}{E(\mathbf{x}-\mathbf{c}_k)(\mathbf{x}-\mathbf{c}_k)} \cdot g_{A,k}^E(\mathbf{x})$$

$$\text{and} \quad f_B^E(\mathbf{x}) = \sum_{k \in B} q_k \delta(\mathbf{x}-\mathbf{c}_k) \cdot g_{B,k}^E(\mathbf{x}),$$

where, q_k is the Coulombic charge on atom k, $\delta(\mathbf{x})$ is the Kronecker delta function with value 1 at $||\mathbf{x}|| = 0$, and 0 everywhere else, $g_{A,k}^E(\mathbf{x}) = g_{B,k}^E(\mathbf{x}) = 1$ and $E(\mathbf{x})$ is the distance dependent dielectric constant [3] as given below.

$$E(\mathbf{x}) = \begin{cases} 4 & \text{if } ||\mathbf{x}|| \le 6\text{Å}, \\ 80 & \text{if } ||\mathbf{x}|| > 8\text{Å}, \\ 38 \cdot ||\mathbf{x}|| - 224 & \text{otherwise.} \end{cases}$$

1.2.1 FFT based formulation

In the current version of F^2Dock 2.0, we define for $P \in A, B$,

$$g_{P,k}^E(\mathbf{x}) = e^{-\beta\left(\frac{(\mathbf{x}-\mathbf{c}_k)^2}{\gamma^2}-1\right)},$$

where, $\gamma > 0$ is a constant, and blobbiness $\beta = 2.3$. In our experiments the new definition of $g^E_{P,k}(\mathbf{x})$ with $\gamma = 3.4$ performed much better than the original Gabb et al. formulation. Smoothing with the Gaussian function as above has the effect of reduced discretization error on the grid.

The overall electrostatics score for translation $\mathbf{t}$ and rotation $\mathbf{r}$ is:

$$H^E_{A,B}(\mathbf{t},\mathbf{r}) = w_E \cdot Re\left(F^E_{A,B}(\mathbf{t},\mathbf{r})\right),$$

where, $F^E_{A,B}(\mathbf{t},\mathbf{r}) = \int_{\mathbf{x}} f^E_A(\mathbf{x}) T_{\mathbf{t}}\left(\Delta_{\mathbf{r}}\left(f^E_B(\mathbf{x})\right)\right)$, and w_E is the user-specified weight given to electrostatics interaction.

1.3 Interface Propensity (IP) and Hydrophobicity (HP).

F^2Dock 2.0 scores the interfaces between molecules A and B using the per-residue interface propensity values computed in [5] based on the relative frequencies of different residues in the interfaces of a set of 63 protein-protein complexes from [6]. Let $IP(R)$ denote the natural logarithm of the interface propensity value of a residue R. Alternatively, the user can choose to use per-residue Hydrophobicity values from [7]. Note that, Hydrophobic residues are more likely to be found on the interface, and hence Hydrophobicity and Interface propensity model the same chemical property and hence are used as substitutes in F^2Dock 2.0, as opposed to using both at the same time.

The IP values for the 20 amino acid residues lie between -0.38 (for ASP) and 0.83 (for TRP). A residue with a higher IP value is likely to occur more frequently in a protein-protein interface than one with a lower IP value. Hydrophobic residues Phe, Met, Ile, Leu and Val, polar aromatic residues Trp, Tyr and His, and the charged residue Arg favor interface locations, and they have positive IP values. Additionally, Cys and Asn also have positive IP. The IP value of an atom is set to the IP value of the residue it belongs to.

Suppose $A + B_{\mathbf{t},\mathbf{r}}$ is a docking pose obtained by rotating molecule B by $\mathbf{r}$ and translating by $\mathbf{t}$. Let $iAtom^+_{\mathbf{t},\mathbf{r}}(P)$ and $iAtom^-_{\mathbf{t},\mathbf{r}}(P)$ denote the set of atoms in the interface of $P \in \{A, B\}$ in this docking pose that have positive and negative IP values, respectively. Also let $iAtom^s_{\mathbf{t},\mathbf{r}}(A,B) = iAtom^s_{\mathbf{t},\mathbf{r}}(A) + iAtom^s_{\mathbf{t},\mathbf{r}}(B)$, for $s \in \{+,-\}$. Then we assign the following interface propensity score to the docking pose:

$$IP\text{-}score_{\mathbf{t},\mathbf{r}}(A,B) = \frac{-\sum_{a \in iAtom^+_{\mathbf{t},\mathbf{r}}(A,B)} IP(a)}{\min\left(IP_\epsilon, \sum_{a \in iAtom^-_{\mathbf{t},\mathbf{r}}(A,B)} IP(a)\right)},$$

where $IP_\epsilon = \max_{IP(R)<0} IP(R)$.

1.3.1 FFT based formulation

We show below how we approximate $IP\text{-}score_{\mathbf{t},\mathbf{r}}(A,B)$. For this approximation, all atoms of A and B are treated as solid spheres.

For $Q \in \{A, B\}$, we define two affinity functions $f^{IP,Q}_A$ and $f^{IP,Q}_B$ for A and B, respectively, and evaluate $F^{IP,Q}_{A,B}(\mathbf{t},\mathbf{r}) = \int_{\mathbf{x}} f^{IP,Q}_A(\mathbf{x}) T_{\mathbf{t}}\left(\Delta_{\mathbf{r}}\left(f^{IP,Q}_B(\mathbf{x})\right)\right)$. Let $Q' \in \{A, B\} \setminus \{Q\}$.

We set the radius of each atom of Q to its van der Waals radius, while the van der Waals radius of each atom of Q' is extended by a non-negative constant r_{ext}[1].

We assign weight $c^{IP,Q}_{P,k}$ to each atom $k \in P = \{Q, Q'\} = \{A, B\}$ as follows:

[1] $r_{ext} = 1.5$Å produced good results in our experiments.

$$c^{IP,Q}_{Q,k} = \begin{cases} IP(k) & \text{if } k \text{ surface atom, } IP(k) > 0, \\ -i \cdot IP(k) & \text{if } k \text{ surface atom, } IP(k) < 0, \\ 0 & \text{otherwise.} \end{cases}$$

$$c^{IP,Q}_{Q',k} = \begin{cases} 1 \text{ if } k \text{ is a surface atom,} \\ 0 \text{ otherwise.} \end{cases}$$

Then we define:

$$f^{IP,Q}_{Q}(\mathbf{x}) = \sum_{k \in Q} c^{IP,Q}_{Q,k} \cdot g^{IP,Q}_{Q,k}(\mathbf{x})$$

where $g^{IP,Q}_{Q,k}(\mathbf{x}) = 1,\ \text{if } |\mathbf{x} - \mathbf{c}_k| \leq r_k$ and 0 otherwise; and

$$f^{IP,Q}_{Q'}(\mathbf{x}) = \bigvee_{k \in Q'} c^{IP,Q}_{Q',k} \cdot g^{IP,Q}_{Q',k}(\mathbf{x})$$

where $g^{IP,Q}_{Q',k}(\mathbf{x}) = 1$ if $|\mathbf{x} - \mathbf{c}_k| \leq r_k + r_{ext}$ and 0 otherwise.

The overall interface propensity score for translation $\mathbf{t}$ and rotation $\mathbf{r}$ (i.e., approximated $IP\text{-}score_{\mathbf{t},\mathbf{r}}(A, B)$) is:

$$H^{IP}_{A,B}(\mathbf{t}, \mathbf{r}) = w_{IP} \cdot \frac{Re\left(F^{IP,A}_{A,B}(\mathbf{t}, \mathbf{r}) + F^{IP,B}_{A,B}(\mathbf{t}, \mathbf{r})\right)}{IP_{\epsilon} + Im\left(F^{IP,A}_{A,B}(\mathbf{t}, \mathbf{r}) + F^{IP,B}_{A,B}(\mathbf{t}, \mathbf{r})\right)},$$

where, w_{IP} is the user-specified weight given to interface propensity.

2 Details on Filters

In this section we describe the implementations of various filters available in F^{2}Dock 2.0. Each filter has a preprocessing step (e.g., construction of octrees and initialization of other relevant data structures) which is executed only once before F^{2}Dock 2.0 enters the rotations loop in phase I, and so not included in the running times of the filters which are executed in each iteration of the loop. Also octrees constructed for the initial configuration of molecule B can be easily transformed on-the-fly so that it correctly matches any rotated configuration of B by applying the corresponding rotation matrix on the node coordinates (e.g., node center) and atoms/points stored in them.

2.1 Lennard-Jones Filter.

We approximate the Lennard-Jones (LJ) potential between molecules A and $B_{\mathbf{t},\mathbf{r}}$ given by the following expression.

$$LJ(A, B_{\mathbf{t},\mathbf{r}}) = \sum_{i \in A, j \in B_{\mathbf{t},\mathbf{r}}} \left(\frac{a_{ij}}{r_{ij}^{12}} - \frac{b_{ij}}{r_{ij}^{6}} \right),$$

where r_{ij} is the distance between atoms $i \in A$ and $j \in B_{\mathbf{t},\mathbf{r}}$, constants a_{ij} and b_{ij} depend on the type (e.g., C, H, O, etc.) of the two atoms involved. For any fixed pair of atom types a_{ij} and b_{ij} are fixed, and are calculated from the Amber force field using well depths μ and equivalence contact distances of homogeneous pairs r_{eqm} as follows (assuming $X = atomType(i \in A)$ and $Y = atomType(j \in B_{\mathbf{t},\mathbf{r}})$) [8, 9].

$$a_{ij} = \mu_{XY} r_{eqm,XY}^{12}, \quad b_{ij} = 2\mu_{XY} r_{eqm,XY}^{6},$$

$$\mu_{XY} = \sqrt{\mu_{XX} + \mu_{YY}}, \; r_{eqm,XY} = \frac{1}{2}(r_{eqm,XX} + r_{eqm,YY})$$

We assume that $X, Y \in \{$C, H, N, O, P, S$\}$.

Our approach is to identify potential docking solutions based on a cutoff potential. Assuming that the docking solution can not have steric clashes at the interface, we can filter (or penalize) all poses with positive LJ potential. However, though this assumption is not unreasonable for bound-bound docking, in the case of bound-unbound and unbound-unbound docking we need to allow some steric clashes. Hence we soften the potential by reducing the $r_{eqm,XX}$ values by a constant factor δ_{LJ} which has the effect of reducing the inter-atomic clash distances[2] by the same factor. As a result docking poses with negative LJ values can now have some clashes, and the maximum number of allowed clashes will depend on the value of δ_{LJ}. In our experiments $\delta_{LJ} = 0.3$ produced good results.

Observe that direct computation of $LJ(A, B_{\mathbf{t},\mathbf{r}})$ requires $\mathcal{O}(M_A M_B)$ time, where M_A (resp. M_B) is the number of atoms in molecule A (resp. B). Instead we use our octree-based fast multipole-type algorithm described in [10] to obtain a $1 + \epsilon$ approximation of the potential in $\mathcal{O}\left(\frac{1}{\epsilon^3}(M_A + M_B)\log(M_A + M_B)\right)$ time and $\mathcal{O}(M_A + M_B)$ space, where $\epsilon \in (0, 1]$ is a user-defined approximation parameter.

[2] i.e., r_{ij} values at which two atoms are considered to be in a clash

2.2 Interface Area Filter.

We sample weighted quadrature/integration points from the surface of each molecule as described in [11, 10]. Sum of the weights of all such integration points is equal to the molecular surface area up to quadrature approximation error, and each integration point can be treated as the center of a small surface patch with its weight being the patch area. Each such patch π with center c_π and area a_π is represented by the tuple $\langle c_\pi, a_\pi \rangle$.

We then evaluate the following expression as an approximation of the interface area between molecules A and $B_{\mathbf{t},\mathbf{r}}$.

$$InterfaceArea(A, B_{\mathbf{t},\mathbf{r}}) = \sum_{\pi \in IP_A(B_{\mathbf{t},\mathbf{r}}) \cup IP_{B_{\mathbf{t},\mathbf{r}}}(A)} a_\pi,$$

where,

$$IP_{P_1}(P_2) = \Big\{\pi_1 | (\pi_1 \in S_{P_1}) \land \exists_{\pi_2 \in S_{P_2}} (dist(c_{\pi_1}, c_{\pi_2}) < \mu_{IA})\Big\}$$

and μ_{IA} is a user-defined constant.

Using our algorithm described in [10] based on octrees [12] and our *Dynamic Packing Grid* (DPG) data structure [13], the expression above can be evaluated in $\mathcal{O}\left((M_A + M_B)\log(M_A + M_B)\right)$ (w.h.p.[3]) time and $\mathcal{O}\left(M_A + M_B\right)$ space. However, this algorithm runs much faster in practice, i.e., in $\mathcal{O}\left(N_{int}\right)$ time (w.h.p.), where N_{int} is the total number of patches in the interface.

2.3 Interface Propensity Filter.

As in Section 2.2 we assume that we have a patch decomposition of the surface of each molecule $P \in \{A, B\}$. However, each patch π is now represented as a triple $\langle c_\pi, a_\pi, h_\pi \rangle$ where the additional value h_π is a measure of interface propensity of π in the following sense. We assume that each atom $k \in P$ is assigned a interface propensity value h_k as in Section 1.3, and $h_\pi = \frac{1}{|\mathcal{S}_{P,\pi}|}\sum_{k \in \mathcal{S}_{P,\pi}} h_k$, where $\mathcal{S}_{P,\pi} = \{k | (k \in P) \land (dist(c_k, c_\pi) < \nu r_k)\}$, c_k is the center of atom k, r_k is its van der Waals radius, and ν is a user-defined constant.

This filter computes $IPVec(A, B_{\mathbf{t},\mathbf{r}})$ which is defined as the following vector:

$$\Bigg\langle \sum_{\substack{\pi \in IP_A(B_{\mathbf{t},\mathbf{r}}) \\ h_\pi < 0}} h_\pi a_\pi, \sum_{\substack{\pi \in IP_A(B_{\mathbf{t},\mathbf{r}}) \\ h_\pi > 0}} h_\pi a_\pi, \sum_{\substack{\pi \in IP_{B_{\mathbf{t},\mathbf{r}}}(A) \\ h_\pi < 0}} h_\pi a_\pi, \sum_{\substack{\pi \in IP_{B_{\mathbf{t},\mathbf{r}}}(A) \\ h_\pi > 0}} h_\pi a_\pi \Bigg\rangle$$

where $IP_{P_1}(P_2)$ is as defined in Section 2.2.

[3]For an input of size n, an event E occurs w.h.p. (with high probability) if, for any $\alpha \geq 1$ and c independent of n, $Pr(E) \leq 1 - \frac{c}{n^\alpha}$.

This vector can be computed simultaneously with the approximate interface area in Section 2.2 incurring negligible overhead. The algorithm is given in [10].

Once we have $IPVec(A, B_{\mathbf{t},\mathbf{r}}) = \langle v_1, v_2, v_3, v_4 \rangle$, we approximate $IP\text{-}score_{\mathbf{t},\mathbf{r}}(A, B)$ defined in Section 1.3 as follows:

$$IP\text{-}score_{\mathbf{t},\mathbf{r}}(A, B) \approx -\frac{v_2 + v_4}{\min\left(IP'_\epsilon, v_1 + v_3\right)},$$

where, $IP'_\epsilon = (\max_{h_k<0} h_k)\,(\min_{\pi \in A \cup B} a_\pi)$.

We also observed that rewarding docking poses with larger positive interface propensity weighted area $v_2 + v_4$ generally improves the ranks of near native poses. However, rewarding based on the product of $IP\text{-}score_{\mathbf{t},\mathbf{r}}(A, B)$ and $v_2 + v_4$ seems to work even better in practice. This composite term which we define as $IP\text{-}product_{\mathbf{t},\mathbf{r}}(A, B)$ below, rewards a docking pose with higher $v_2 + v_4$ value more than one with lower such value when both have the same $IP\text{-}score$.

$$\begin{aligned} IP\text{-}product_{\mathbf{t},\mathbf{r}}(A, B) &= IP\text{-}score_{\mathbf{t},\mathbf{r}}(A, B) \times (v_2 + v_4) \\ &\approx -\frac{(v_2 + v_4)^2}{\min\left(IP'_\epsilon, v_1 + v_3\right)} \end{aligned}$$

The interface propensity filter in F^2Dock 2.0 penalizes a docking pose provided its *IP-score* is below a user-defined threshold μ_{IP}. The *IP-product* value, after weighting it by a user-specified weight w'_{IP}, is added to the overall score of the pose.

2.4 Clash Filter.

Two atoms $a \in A$ and $b \in B$ with van der Waals radii r_a and r_b, respectively, are said to be in a *clash* provided the distance between their centers is smaller than $\alpha(r_a + r_b)$, where α is a user-defined positive constant. F^2Dock 2.0 counts the total number of atomic clashes between molecules A and $B_{\mathbf{t},\mathbf{r}}$ as follows.

$$Clash_{\mathbf{t},\mathbf{r}}(A, B) = \left|\{b | (b \in B_{\mathbf{t},\mathbf{r}}) \wedge \exists_{a \in A}(r_{a,b} < \alpha(r_a + r_b))\}\right|$$

Direct computation of this number requires $\mathcal{O}(M_A M_B)$ time. So we use an octree-based spatial subdivision approach that is similar to the interface area approximation algorithm used in Section 2.2 and has similar running time.

F^2Dock 2.0 penalizes a docking pose $A + B_{\mathbf{t},\mathbf{r}}$ provided $Clash_{\mathbf{t},\mathbf{r}}(A, B) > \mu_C$, where μ_C is a user-defined constant. A value of around 10 for μ_C seems to work well in practice.

2.5 Residue-Residue Contact Filter.

F^2Dock 2.0 uses the pairwise contact preferences listed in either Table III (without volume normalization) or Table IV (normalized w.r.t. residue volumes) of [14] based on user preference with Table III being the default. The contact preferences were derived as follows. Two residues are considered to be in contact if the distance between their C_β atoms (C_α for Gly) is less than 6 Å. The normalized number of contacts between residue types i and j is defined as $Q_{ij} = C_{ij} / \sum_{k<l} C_{kl}$, where C_{ij} is the actual number of contacts observed between residue types i and j. The expected number of contacts W_{ij} is obtained by assuming that there are no contact preferences between residues of

different types. Then the likelihood of contact between residues types i and j is defined as (Table III of [14]): $G_{ij} = 10 \log \frac{Q_{ij}}{W_{ij}}$.

The volume-normalized likelihood $G_{ij}(v)$ (Table IV of [14]) is obtained by replacing Q_{ij} with: $Q_{ij}(v) = C_{ij} \times V_i \times V_j / \sum_{k<l} (C_{kl} \times V_k \times V_l)$, where V_i is the volume of residue i.

Given a docking pose $A + B_{\mathbf{t},\mathbf{r}}$, we identify all residue-residue contacts at the interface of the two molecules using a fast algorithm similar to the one used in Section 2.3, and compute the sum of all positive and negative G_{ij} values (or $G_{ij}(v)$ values if chosen by the user) denoted by G^+ and G^-, respectively. Then we compute the following ratio:

$$RC\text{-}score_{\mathbf{t},\mathbf{r}}(A, B) = -\frac{G^+}{\min(G^-, RC_\epsilon)},$$

where $RC_\epsilon = \max_{G_{ij}<0} G_{ij}$ (or $RC_\epsilon = \max_{G_{ij}(v)<0} G_{ij}(v)$ if the user chooses volume-normalized contact preferences).

F^{2}Dock 2.0 penalizes a docking pose if its *RC-score* is below a user-specified constant $\mu_R C$. Without volume normalization $\mu_R C = 3.0$ seems to work well in practice. Docking poses can also be penalized based on user-specified lower and upper bounds for G^+ and G^-, respectively.

2.6 Glycine Filter.

We mark the oligopeptides with the properties described above on the enzyme surface, and for any given docking pose count the number of these motifs occurring at the interface using a fast algorithm similar to the one used for counting atomic clashes in Section 2.4.

2.7 Antibody-Antigen Contact Filter.

We say that an atom a is in *close neighborhood* of another atom b provided the distance between the centers of a and b is at most $2(r_a + r_b)$. Given a potential antibody-antigen docking pose, F^{2}Dock 2.0 computes three quantities: $N_{L1\cup H1}$, N_{L3} and N_{H3}, denoting the number of antigen atoms that are in the close neighborhood of any atom in the antibody regions CDR-L1/CDR-H1, CDR-L3 and CDR-H3, respectively. These counts are obtained using a fast algorithm similar to the one used in Section 2.4. F^{2}Dock 2.0 penalizes a docking pose provided $N_{L1\cup H1} < \mu_{L1\cup H1}$ or $N_{L3} < \mu_{L3}$ or $N_{H3} < \mu_{H3}$, otherwise it adds $w_{AC}(N_{L1\cup H1} + N_{L3} + N_{H3})$ to the total score, where $\mu_{L1\cup H1}$, μ_{L3}, μ_{H3} and w_{AC} are user-specified quantities. F^{2}Dock 2.0 uses $\mu_{L1\cup H1} = 100$, $\mu_{L3} = \mu_{H3} = 150$ and $w_{AC} = 10$ by default. The CDR (Complementarity Determining Region) loops are identified using the method described in [15].

2.8 Overall Cost of Filtering.

Summing up the cost of all filters described above for a single run, we obtain
$\mathcal{O}\left(\frac{1}{\epsilon^3}(M_A + M_B)\log(M_A + M_B)\right)$ (w.h.p).
Assuming that each filter is applied on at most N_F configurations, the total time taken by all filters is
$\mathcal{O}\left(\frac{1}{\epsilon^3}N_F(M_A + M_B)\log(M_A + M_B)\right)$ (w.h.p.).
Assuming $N_F = \mathcal{O}(N_R)$, where N_R is the number of samples in the rotations space, the running time reduces to
$\mathcal{O}\left(\frac{1}{\epsilon^3}N_R(M_A + M_B)\log(M_A + M_B)\right)$ (w.h.p.).

3 Solvation Energy Based Reranking

The solvation energy E_{sol} consists of the energy to form cavity in the solvent (E_{cav}), the solute-solvent van der Waals interaction energy ($E_{\text{vdw(s-s)}}$), and the electrostatic potential energy change due to the solvation (also known as the polarization energy, E_{pol}) [16, 17, 18, 19].

$$E_{\text{sol}} = \underbrace{E_{\text{cav}} + E_{\text{vdw(s-s)}}}_{\text{nonploar}} + \underbrace{E_{\text{pol}}}_{\text{polar}}$$

The first two terms are often modeled as [16, 20]

$$E_{\text{cav}} = pV + \sum_i \gamma_i A_i$$

and

$$E_{\text{vdw(s-s)}} = \rho_0 \sum_i \int_{\text{ex}} u_i^{(\text{att})}(\mathbf{x}_i, \mathbf{r}) d^3\mathbf{r}$$

where p is the solvent pressure, V is the molecular volume, A_i is the solvent accessible surface area of atom i and γ_i is its solvation parameter, ρ_0 is the bulk density, and $u_i^{(\text{att})}$ is the van der Waals dispersive component of the interaction between atom i and the solvent.

The last term, E_{pol}, can be approximated using *Generalized Born* (GB) theory [21].

$$E_{\text{pol}} = -\frac{\tau}{2} \sum_{i,j} \frac{q_i q_j}{\sqrt{r_{ij}^2 + R_i R_j e^{-\frac{r_{ij}^2}{4R_iR_j}}}}, \tag{1}$$

where $\tau = 1 - \frac{1}{\epsilon}$, and R_i is the effective Born radius of atom i.

GB-rerank approximates each of these terms as described in the following sections, and reranks the list of top docking poses produced by F^{2}Dock 2.0 based on the resulting ΔE_{sol} values. In order to approximate ΔE_{sol}, GB-rerank precomputes the E_{sol} values for molecules A and B, and then computes E_{sol} for each docking pose.

3.1 Approximating E_{pol} (Polarization Energy).

We first estimate all Born radii of the molecule/complex using our fast approximation scheme described in [10] followed by another of our algorithms from [10] for estimating E_{pol} from the approximated Born radii. Given a molecule/complex consisting of M atoms, both algorithms run in $\mathcal{O}\left(\frac{1}{\epsilon^3} M \log M\right)$ time using $\mathcal{O}(M)$ space, where $\epsilon > 0$ is an approximation parameter that provides a speed-accuracy tradeoff. The smaller the value of ϵ the more accurate the algorithms are, and the larger the ϵ value the faster they run.

GB-rerank uses either the r^4- or the r^6-approximation of R_i (see [10]) based on user preference. By default, it uses the r^6-approximation [22] which shows better accuracy for spherical solutes as well as for proteins [23].

$$\frac{1}{R_i^3} = \frac{3}{4\pi} \int_{\text{ex}} \frac{1}{|\mathbf{r} - \mathbf{x}_i|^6} d^3\mathbf{r}, \quad i \in [1, M]. \tag{2}$$

where Γ is the molecular surface, $\mathbf{n}(\mathbf{r})$ is the unit outward normal on the molecular surface at $\mathbf{r}$, and $\mathbf{x}_i$ is the center of atom i.

We obtain the following discrete surface formulation of Equation (2) by applying the divergence theorem and Gaussian quadrature.

$$\frac{1}{{R_i}^3} \approx \frac{1}{4\pi}\sum_{k=1}^{N} w_k \frac{(\mathbf{r}_k - \mathbf{x}_i)\cdot \vec{\mathbf{n}}_k}{|\mathbf{r}_k - \mathbf{x}_i|^6}. \tag{3}$$

where, the $\mathbf{r}_k$'s are $N = \mathcal{O}(M)$ Gauss quadrature/integration points [11] on the molecular surface, and w_k is a weight assigned to $\mathbf{r}_k$ in order to achieve higher order of accuracy for small N.

In order to avoid the slowdown due to the repeated computation of quadrature points for each docking pose $A + B_{\mathbf{t},\mathbf{r}}$, F^2Dock 2.0 precomputes the set of quadrature points Q_A and Q_B for A and B, respectively, and chooses all quadrature points from $Q_A \cup Q_{B_{\mathbf{t},\mathbf{r}}}$ that are not on the interface of A and $B_{\mathbf{t},\mathbf{r}}$ for use during the Born radii estimation of $A + B_{\mathbf{t},\mathbf{r}}$.

3.2 Approximating $E_{\text{vdw(s-s)}}$ (Dispersion Energy).

The solute-solvent van der Waals interaction energy (also known as *dispersion energy*) is modeled as [16, 20]:

$$E_{\text{vdw(s-s)}} = \rho_0 \sum_{i=1}^{M} \int_{\text{ex}} u_i^{(\text{att})}(\mathbf{x}_i, \mathbf{r}) d^3\mathbf{r}.$$

where ρ_0 is the bulk density, and $u_i^{(\text{att})}$ is the van der Waals dispersive component of the interaction between atom $i \in [1, M]$ and the solvent which is given as follows.

$$u_i^{(\text{att})}(\mathbf{x}_i, \mathbf{r}) = \frac{1}{|\mathbf{r} - \mathbf{x}_i|^6}$$

Thus

$$E_{\text{vdw(s-s)}} = \rho_0 \sum_{i=1}^{M} \int_{\text{ex}} \frac{1}{|\mathbf{r} - \mathbf{x}_i|^6} d^3\mathbf{r} \tag{4}$$

If R_i is the Born radius of atom i calculated using the r^6-approximation (i.e., Equation 2/3), then Equation 4 can be rewritten as:

$$E_{\text{vdw(s-s)}} \approx \rho_0 \frac{4\pi}{3} \sum_{i=1}^{M} \frac{1}{R_i^3} \tag{5}$$

Therefore, $E_{\text{vdw(s-s)}}$ can be approximated in $\mathcal{O}(M)$ time once the Born radii of all atoms are available.

3.3 Approximating E_{cav} (Cavity Forming Energy).

Instead of computing E_{cav} for A, B and each $A + B_{\mathbf{t},\mathbf{r}}$ separately, we approximate ΔE_{cav} with the buried surface area of $A + B_{\mathbf{t},\mathbf{r}}$ which is approximated using the same algorithm used for interface area filter in Section 2.2. The algorithm runs in $\mathcal{O}((M_A + M_B)\log(M_A + M_B))$ (w.h.p.) time (much faster in practice; see Section 2.2) using $\mathcal{O}(M_A + M_B)$ space.

3.4 Overall Cost of Reranking.

Assuming that GB-rerank is applied on N_G docking poses, its total running time is
$\mathcal{O}\left(\frac{1}{\epsilon^3} N_G (M_A + M_B) \log(M_A + M_B)\right)$ (w.h.p.).
Typically $N_G = \mathcal{O}(N_R)$, where N_R is the number of samples in the rotations space, and so the running time reduces to
$\mathcal{O}\left(\frac{1}{\epsilon^3} N_R (M_A + M_B) \log(M_A + M_B)\right)$ (w.h.p.).

4 Additional Results on the Effects of Various Affinity Functions and Filters

Figures S3 to S10 track the changes in the rank of the top hit, RMSD of the top hit, number of hits in top 1000, and the minimum RMSD in the top 1000 predictions, respectively, as the various F^2Dock 2.0 options are activated one after another on the rigid-body test cases from Zlab benchmark 2.0 [2].

When we activate Lennard-Jones filter, clash filter and proximity clustering after shape complementarity we get hits for 4 new test cases, and the rank of the top hit improves for 15 more (see Figure S3(top)). However, we also lose hits in top 1000 for 3 test cases, and the rank of the top hit degrades for one test case. Overall, the application of these filters and clustering seem largely beneficial. The best results are obtained for enzyme-inhibitor/enzyme-substrate complexes, as for more than 50% of these complexes rank of the top hit improve.

The impact on the RMSD of the top hit is not clear though it has improved for 5 test cases in the enzyme-inhibitor/enzyme-substrate group (see Figure S5(top)). Similar is the impact on the minimum RMSD in the top 1000 predictions (see Figure S9(top)).

The number of hits in the top 1000 predictions is reduced in majority of the cases (see Figure S7(top)) as the two filters penalize predictions that are close to the target complex but have steric clashes. The proximity clustering step also penalizes poses that are very similar to poses with higher scores.

When electrostatics is turned on we get hits in top 1000 for 9 test cases for which we did not have a single hit before, and for 14 other cases rank of the top hit improve (see Figure S3(middle)). However, we lose all hits for 1 test case, and for 4 others rank of the top hit degrades.

Though there does not seem to be any particular trend in the change of the RMSD of the top hit except for the 9 cases with new first hits as mentioned above (see Figure S5(middle)), for antibody-antigen and antigen-bound antibody complexes the minimum RMSD in the top 1000 seems to have generally improved (see Figure S9(middle)). For 22 test cases the number of hits in the top 1000 predictions has improved while for 5 cases this number has dropped (see Figure S7(middle)).

The FFT-based interface propensity scoring is activated next which improves the rank of the top hit for 30 test cases (i.e., for around 50% of all cases) among which 7 cases did not have a single hit before (see Figure S3(bottom)). Among these 7 cases with new first hits 5 are antibody-antigen or antigen-bound antibody complexes, and none are enzyme-inhibitor or enzyme-substrate. Ranks have degraded for 5 test cases. Minimum RMSD in top 1000 has improved for 20 test cases degrading for only 1 (see Figure S9(bottom)), and RMSD of top hit has also degraded for only 1 test case (see Figure S5(bottom)). The number of hits has increased for 28 test cases and decreased for 7 (see Figure S7(bottom)).

The interface propensity filter is turned on next. It improves the rank of the top hit for 25 complexes, and degrades for 5 (see Figure S4(top)). For 3 test cases we did not have a single hit in top 1000 before among which 2 are antibody-antigens. The number of hits has increased for 16 cases, and decreased for 5 (see Figure S8(top)). The impacts on the RMSD of the top hit and the minimum RMSD in top 1000 are not significant in general (see Figures S6(top) and S10(top)).

The residue-residue contact filter which is activated next improves the rank of the top hit for 27 test cases, and degrades for none (see Figure S4(middle)). The enzyme-inhibitor and enzyme-substrate complexes seem to have benefited the least from this filter. The impact on the number of hits in top 1000 is not significant and is mixed (see Figure S8(middle)). The RMSD of the top hit

and the minimum RMSD in top 1000 do not change except for the 3 cases for which we get new first hits (see Figures S6(middle) and S10(middle)).

Next we apply the antibody contact filer and the Glycine filter. The antibody contact filter improves the rank of the top hit for 9 antibody-antigen and antigen-bound antibody test cases, and degrades for 3, while the Glycine filter slightly improves the same for 4 enzyme-inhibitor/enzyme-substrate complexes (see Figures S4(bottom)). The antibody contact filter improves the number of hits in the top 1000 predictions for 5 complexes (see Figure S8(bottom)).

All other changes are insignificant.

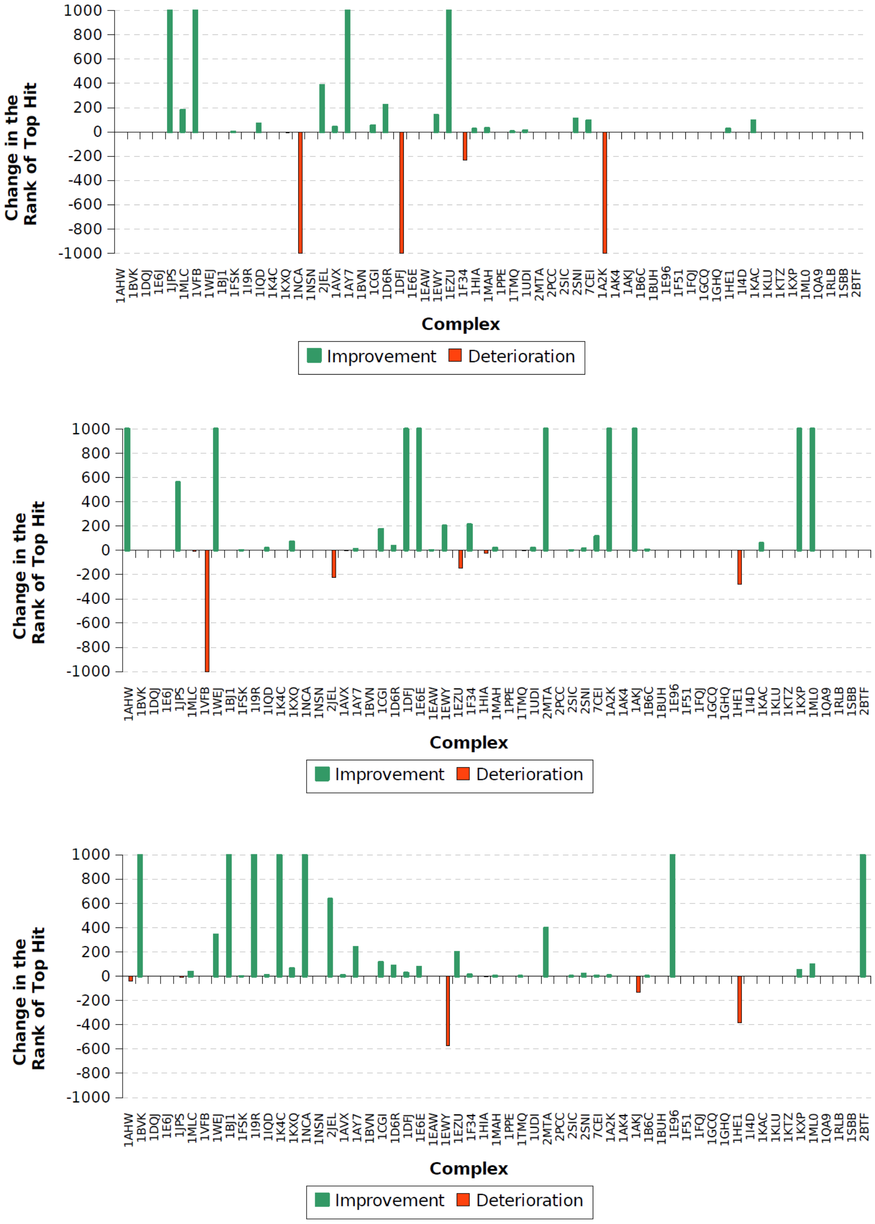

Figure S3: **Changes in the rank of top hit as various options in F^{2}Dock 2.0 are activated one after another** (on the rigid-body test cases from Zlab benchmark 2.0 [2]). **Top:** Lennard-Jones Filter (LJ), clash filter (CL) and proximity clustering (PC) are activated after shape complementarity (SC), **Middle:** electrostatics & charge complementarity (EL) after SC+LJ+CL+PC, **Bottom:** interface propensity (IP) after SC+LJ+CL+PC+EL.

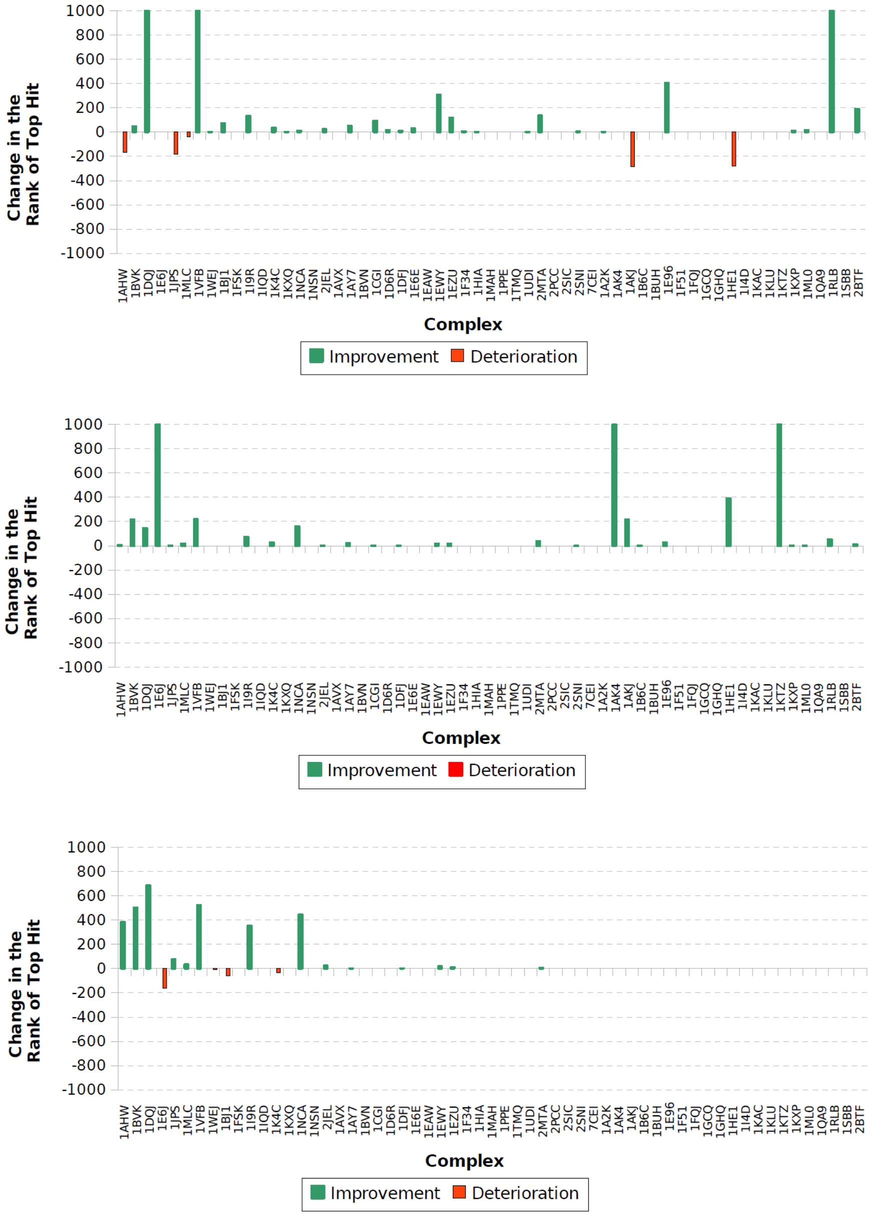

Figure S4: **Changes in the rank of top hit as various options in F^2Dock 2.0 are activated one after another** (on the rigid-body test cases from Zlab benchmark 2.0 [2]). Continued from S3. **Top:** interface propensity filter (PF) after SC+LJ+CL+PC+EL+IP, **Middle:** residue-residue contact filter (RC) after SC+LJ+CL+PC+EL+IP+PF, and **Bottom:** antibody contact filter (AF) or glycine filter (GF) after SC+LJ+CL+PC+EL+IP+PF+RC.

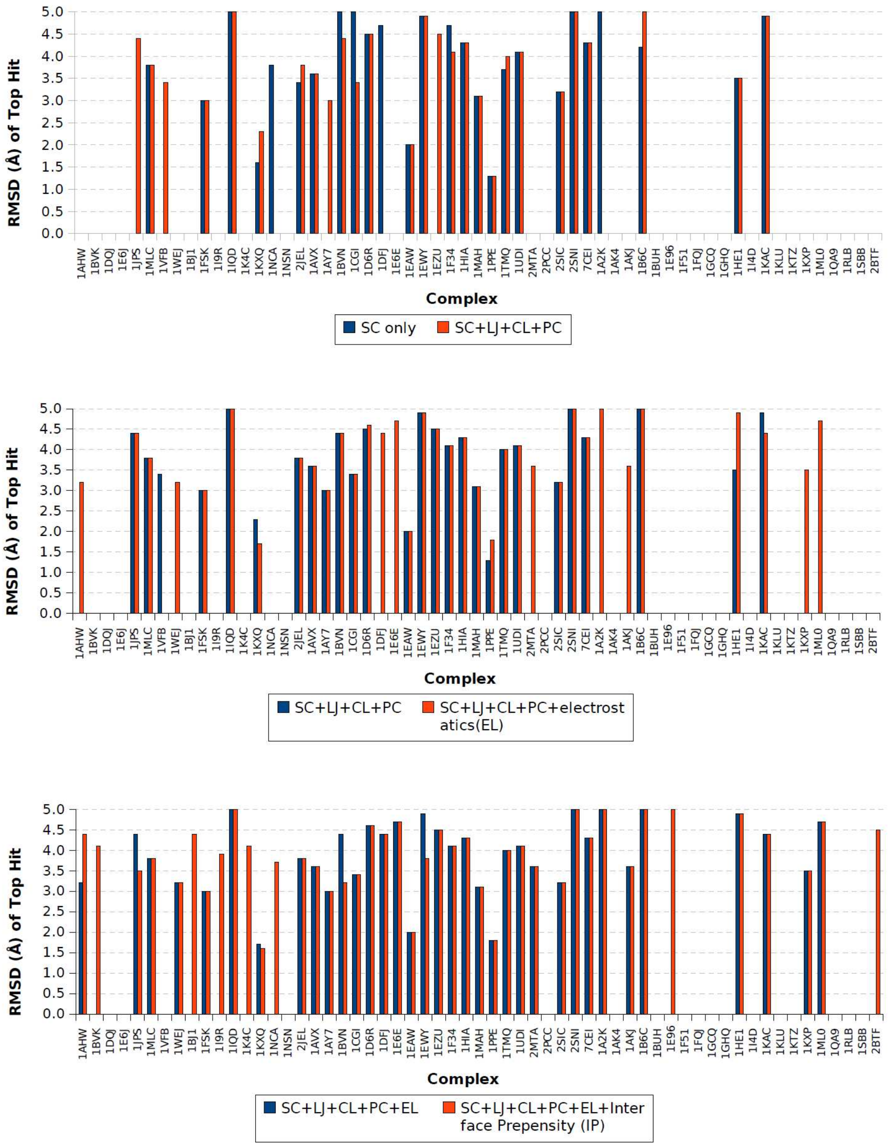

Figure S5: **Changes in the RMSD of the top hit as various options in F^{2}Dock 2.0 are activated one after another** (on the rigid-body test cases from Zlab benchmark 2.0 [2]). **Top:** Lennard-Jones Filter (LJ), clash filter (CL) and proximity clustering (PC) are activated after shape complementarity (SC), **Middle:** electrostatics & charge complementarity (EL) after SC+LJ+CL+PC, **Bottom:** interface propensity (IP) after SC+LJ+CL+PC+EL.

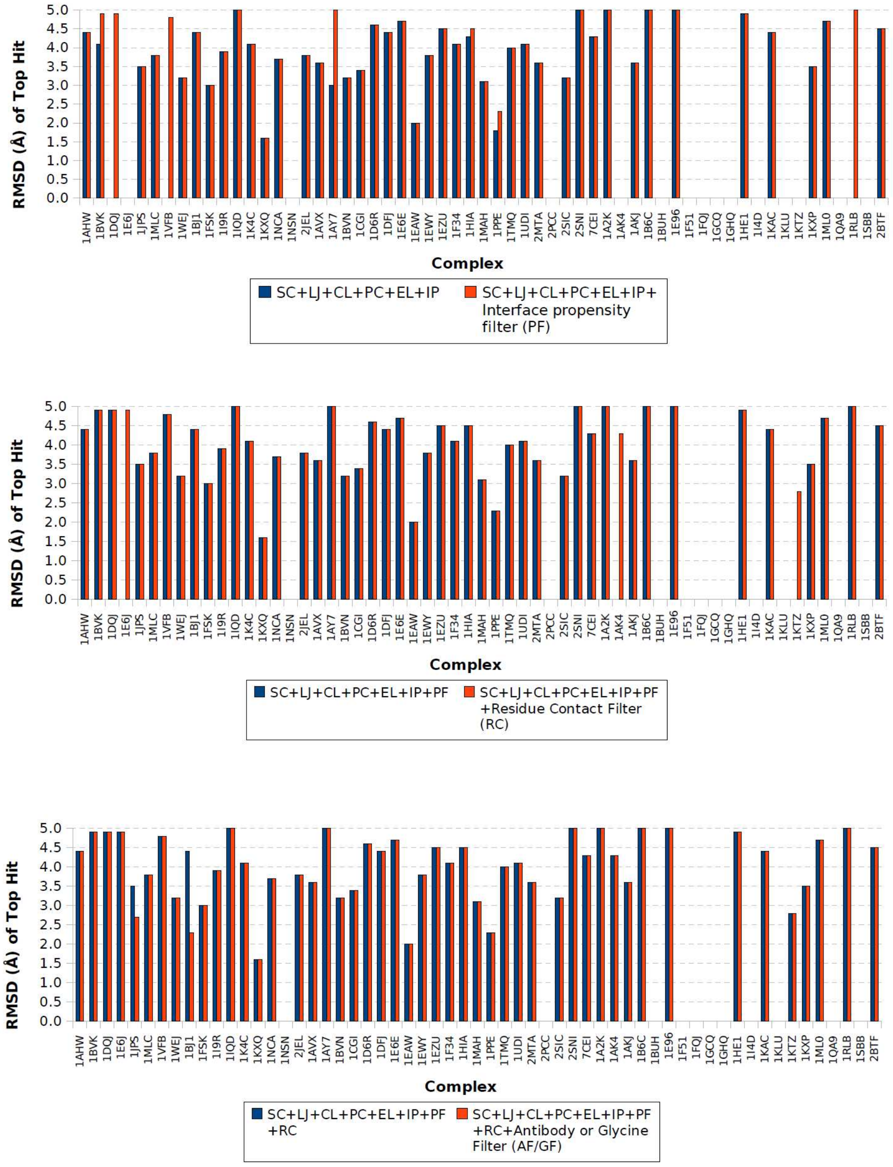

Figure S6: **Changes in the RMSD of the top hit as various options in F^{2}Dock 2.0 are activated one after another** (on the rigid-body test cases from Zlab benchmark 2.0 [2]). Continued from S5. **Top:** interface propensity filter (PF) after SC+LJ+CL+PC+EL+IP, **Middle:** residue-residue contact filter (RC) after SC+LJ+CL+PC+EL+IP+PF, and **Bottom:** antibody contact filter (AF) or glycine filter (GF) after SC+LJ+CL+PC+EL+IP+PF+RC.

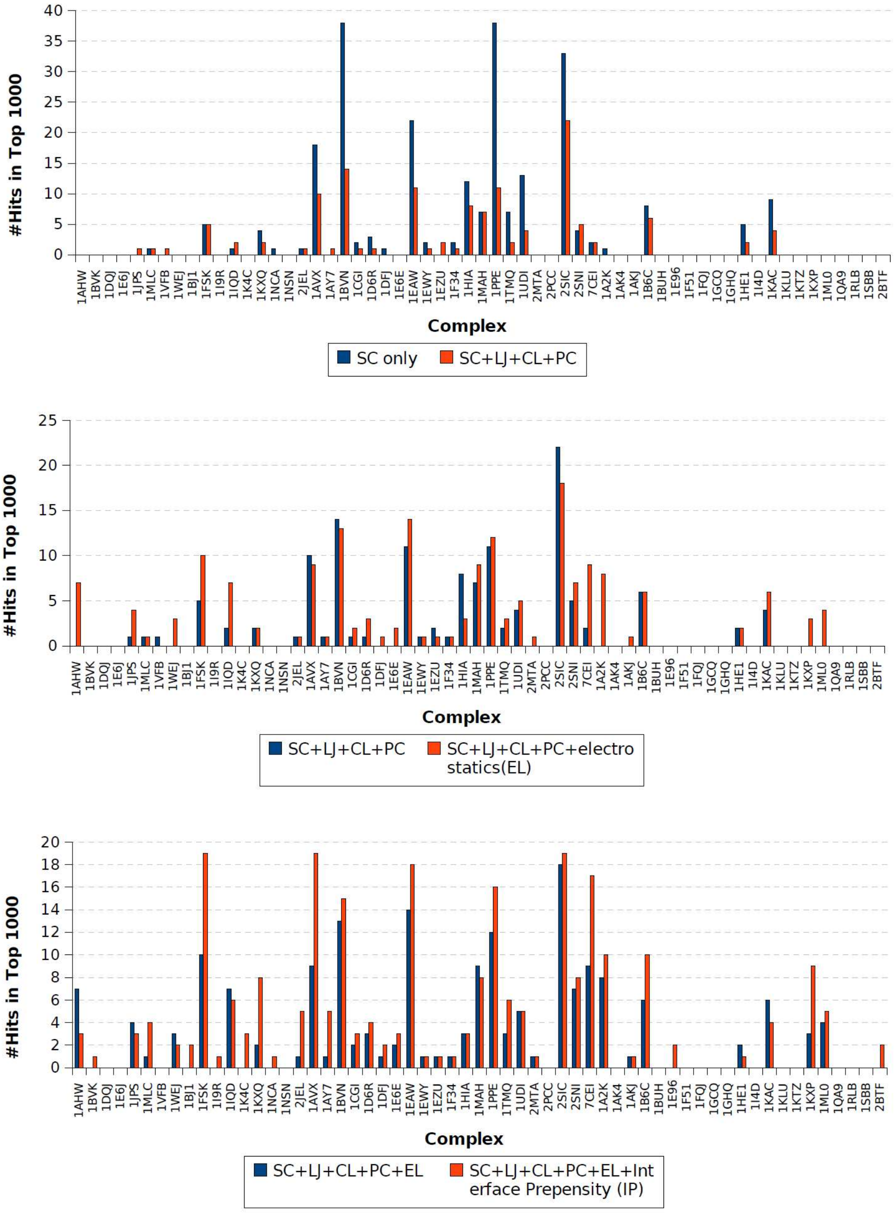

Figure S7: **Changes in the number of hits in the top 1000 docking poses generated by F^2Dock 2.0 as various options in F^2Dock 2.0 are activated one after another** (on the rigid-body test cases from Zlab benchmark 2.0 [2]). **Top:** Lennard-Jones Filter (LJ), clash filter (CL) and proximity clustering (PC) are activated after shape complementarity (SC), **Middle:** electrostatics & charge complementarity (EL) after SC+LJ+CL+PC, **Bottom:** interface propensity (IP) after SC+LJ+CL+PC+EL.

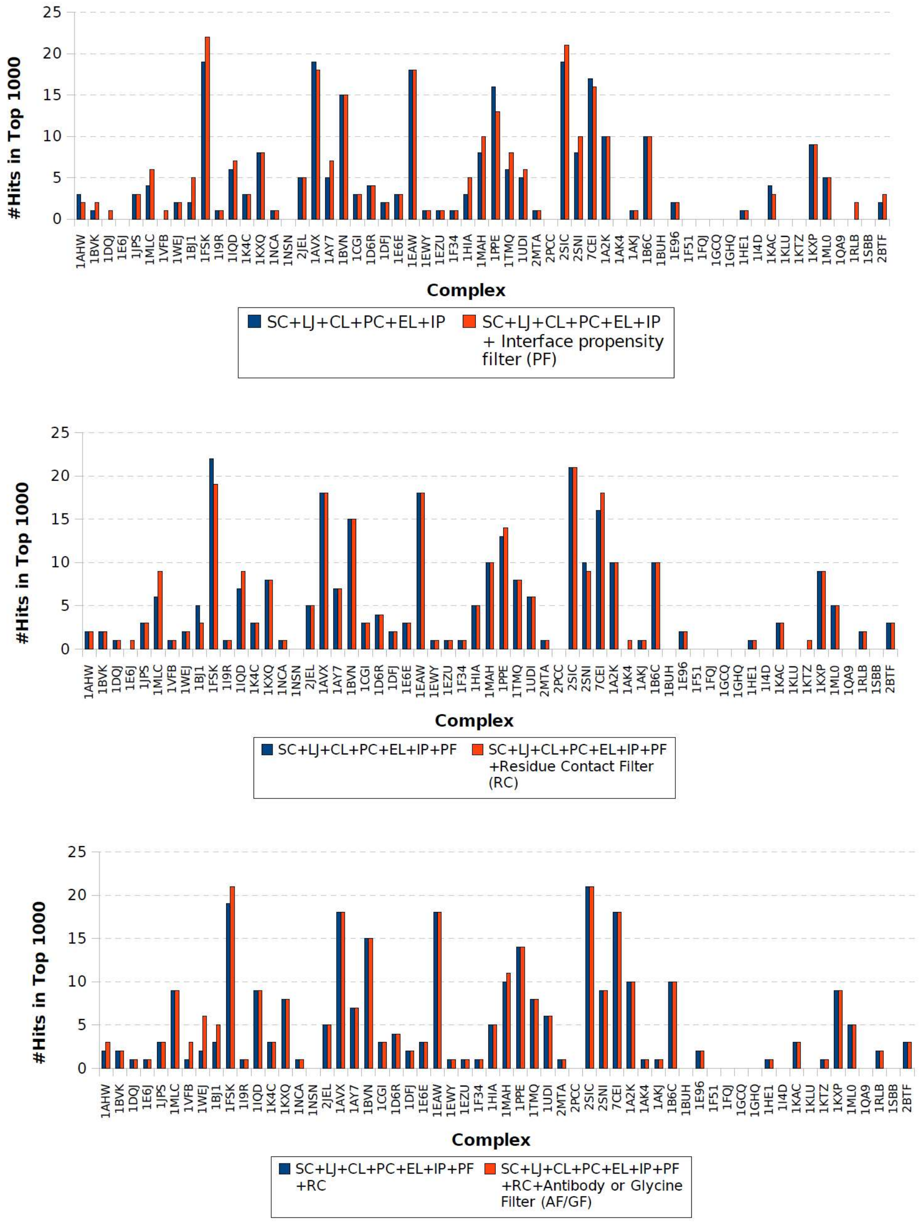

Figure S8: **Changes in the number of hits in the top 1000 docking poses generated by F^{2}Dock 2.0 as various options in F^{2}Dock 2.0 are activated one after another** (on the rigid-body test cases from Zlab benchmark 2.0 [2]). Continued from S7. **Top:** interface propensity filter (PF) after SC+LJ+CL+PC+EL+IP, **Middle:** residue-residue contact filter (RC) after SC+LJ+CL+PC+EL+IP+PF, and **Bottom:** antibody contact filter (AF) or glycine filter (GF) after SC+LJ+CL+PC+EL+IP+PF+RC.

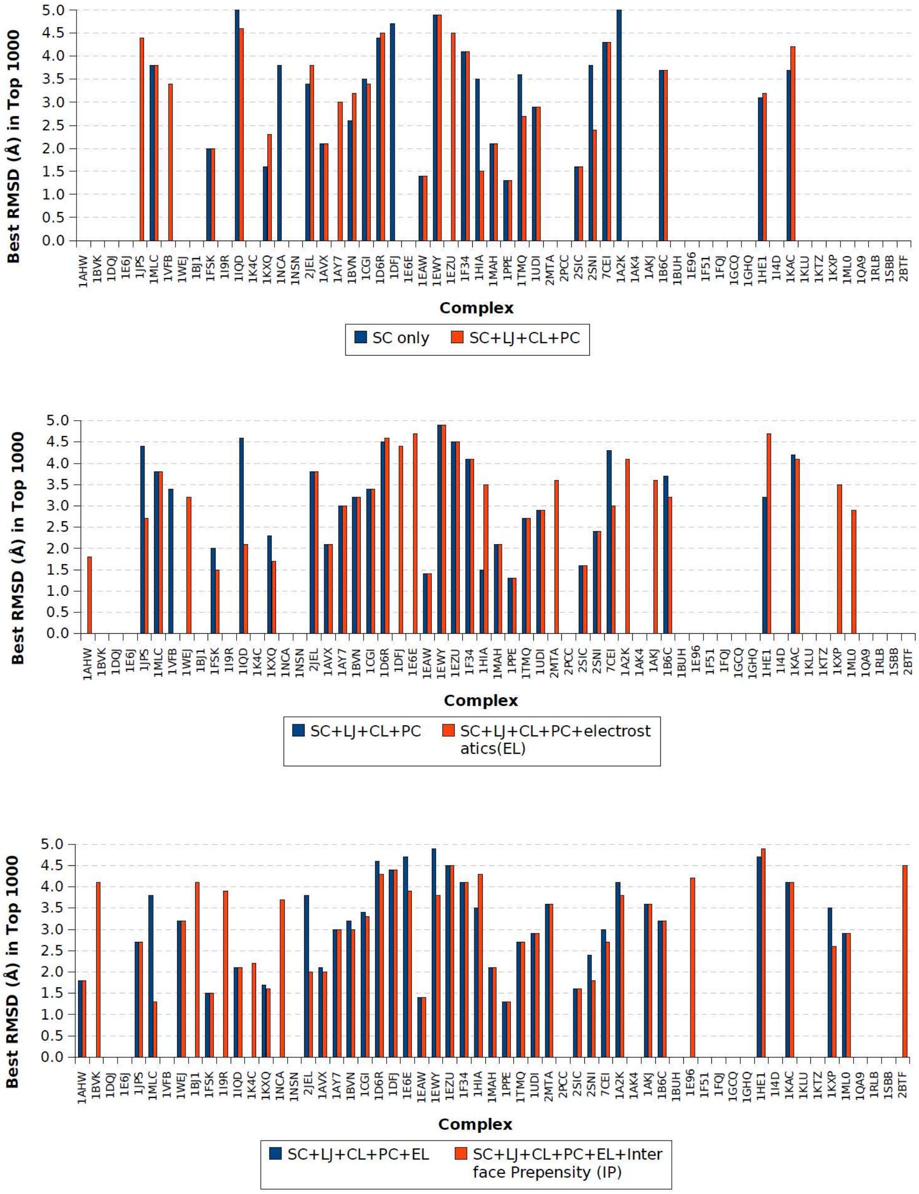

Figure S9: **Changes in the minimum RMSD in the top 1000 docking poses generated bt F^2Dock 2.0 as various options in F^2Dock 2.0 are activated one after another** (on the rigid-body test cases from Zlab benchmark 2.0 [2]). **Top:** Lennard-Jones Filter (LJ), clash filter (CL) and proximity clustering (PC) are activated after shape complementarity (SC), **Middle:** electrostatics & charge complementarity (EL) after SC+LJ+CL+PC, **Bottom:** interface propensity (IP) after SC+LJ+CL+PC+EL.

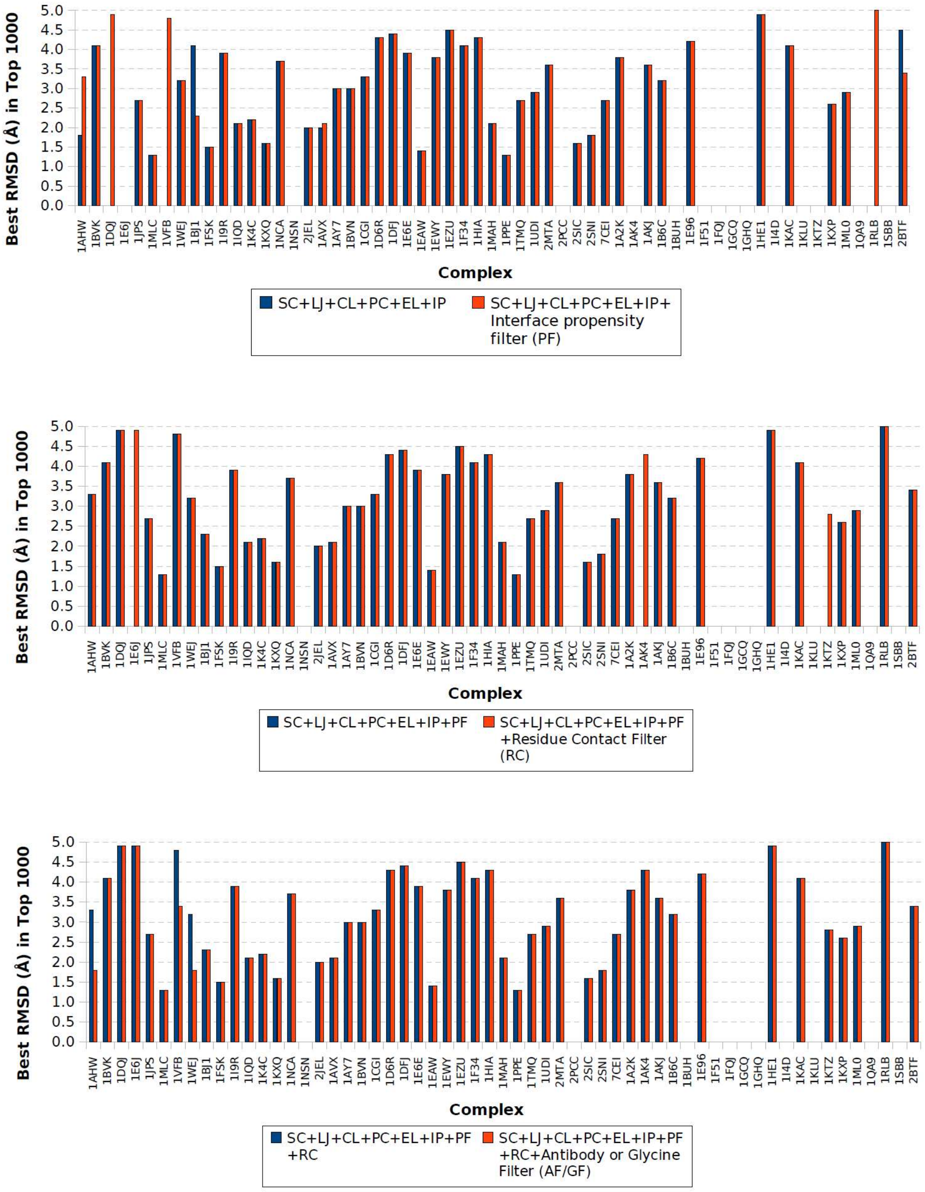

Figure S10: **Changes in the minimum RMSD in the top 1000 docking poses generated bt F^{2}Dock 2.0 as various options in F^{2}Dock 2.0 are activated one after another** (on the rigid-body test cases from Zlab benchmark 2.0 [2]). Continued from S9. **Top:** interface propensity filter (PF) after SC+LJ+CL+PC+EL+IP, **Middle:** residue-residue contact filter (RC) after SC+LJ+CL+PC+EL+IP+PF, and **Bottom:** antibody contact filter (AF) or glycine filter (GF) after SC+LJ+CL+PC+EL+IP+PF+RC.

5 Additional Results Comparing ZDock 3.0.2 and F^2Dock 2.0

Class	Difficulty	Complex	Top ranked near-native solution								Best ranked near-native solution							
			ZDock				F2Dock				ZDock				F2Dock			
			Rank	RMSD	F_nat	Verdict	Rank	RMSD	F_nat	Verdict	Rank	RMSD	F_nat	Verdict	Rank	RMSD	F_nat	Verdict
Antibody	Easy	1AHW	354	4.5	0.30	Acc	8	4.4	0.47	Acc	1242	0.9	0.77	High	457	1.8	0.69	Med
		1BJ1	1	1.9	0.69	Med	63	2.3	0.53	Acc	1	1.9	0.69	Med	63	2.3	0.53	Acc
		1BVK	184	3.6	0.54	Med	205	4.9	0.23	Acc	358	1.9	0.67	Med	264	4.1	0.35	Acc
		1DQJ	374	4.0	0.56	Acc	74	4.9	0.21	Acc	1787	3.3	0.44	Med	74	3.6	0.21	Acc
		1E6J	3	4.1	0.20	Acc	126	5	0.19	Acc	181	2.7	0.58	Med	126	5	0.19	Acc
		1FSK	1	2.9	0.52	Med	1	3.2	0.46	Acc	2	1.8	0.58	Med	3	1.5	0.71	Med
		1I9R					9	3.9	0.42	Acc					9	3.9	0.42	Acc
		1IQD	18	4.3	0.48	Acc	4	3	0.38	Med	68	1.7	0.68	Med	4	3	0.38	Med
		1JPS	1266	2.1	0.65	Med	186	2.7	0.61	Acc	1266	2.1	0.65	Med	186	2.7	0.61	Acc
		1K4C	583	2.9	0.56	Med	105	4.4	0.47	Acc	583	2.9	0.56	Med	165	2.2	0.61	Med
		1KXQ	2	1.2	0.78	Med	1	1.6	0.75	Med	2	1.2	0.78	Med	1	1.6	0.75	Med
		1MLC	57	2.0	0.41	Acc	11	3.8	0.32	Acc	57	2.0	0.41	Acc	114	1.3	0.65	Med
		1NCA	11	1.7	0.56	Med	168	3.7	0.55	Acc	11	1.7	0.56	Med	168	3.7	0.55	Acc
		1NSN	1267	1.6	0.67	Med					1267	1.6	0.67	Med				
		1QFW					80	1.9	0.57	Med					80	1.9	0.57	Med
		1VFB	250	3.1	0.43	Acc	191	4.8	0.34	Acc	560	2.9	0.46	Med	434	3.4	0.42	Med
		1WEJ	9	1.5	0.75	Med	5	3.2	0.65	Med	9	1.5	0.75	Med	5	3.2	0.65	Med
		2FD6	3	5.0	0.18	Inc	62	4.4	0.26	Acc	282	3.3	0.25	Acc	62	4.4	0.26	Acc
		2I25	2	3.0	0.55	Med	122	3.9	0.51	Med	40	1.7	0.54	Med	242	2.6	0.49	Med
		2JEL	4	3.5	0.50	Med	1	3.3	0.45	Med	753	2.6	0.56	Med	1	3.3	0.45	Med
		2VIS																
		9QFW	2	4.0	0.67	Inc	1	3.9	0.31	Acc	48	1.9	0.71	Med	3	2.9	0.51	Med
	Medium	1BGX																
	Hard	1E4K																
		2HMI																

Figure S11: Comparison of the performance of F^2Dock 2.0 and ZDock 3.0.2 for each of the 25 antibody-antigen and antigen-bound antibody complexes from ZLab's benchmark 4.0 in terms of the rank, RMSD, F_{nat} and CAPRI classification of the top hit and the best hit.

Class	Difficulty	Complex	Top ranked near-native solution								Best ranked near-native solution							
			ZDock				F2Dock				ZDock				F2Dock			
			Rank	RMSD	F_nat	Verdict	Rank	RMSD	F_nat	Verdict	Rank	RMSD	F_nat	Verdict	Rank	RMSD	F_nat	Verdict
Enzyme	Easy	1AVX	25	3.5	0.60	Med	1	4.5	0.40	Acc	194	1.5	0.76	Med	4	2	0.83	Med
		1AY7	577	2.5	0.67	Med	2	4	0.36	Med	577	2.5	0.67	Med	6	2.5	0.63	Med
		1BVN	3	1.2	0.78	Med	1	3.2	0.55	Med	3	1.2	0.78	Med	2	3	0.54	Med
		1CGI	10	4.0	0.54	Acc	76	3.4	0.53	Med	173	2.6	0.64	Med	199	3.3	0.56	Med
		1CLV	3	2.3	0.76	Med	1	2.5	0.64	Med	21	2.3	0.63	Med	350	2.1	0.79	Med
		1D6R					59	4.6	0.28	Acc					249	4.3	0.41	Acc
		1DFJ	1	4.1	0.43	Acc	9	4.4	0.58	Acc	2	3.2	0.57	Med	9	4.4	0.58	Acc
		1E6E	5	3.2	0.49	Med	20	4.7	0.37	Med	10	1.5	0.74	Med	20	3.9	0.37	Med
		1EAW	68	3.4	0.56	Med	1	1	0.84	Med	579	1.7	0.72	Med	1	1	0.84	Med
		1EWY	53	4.2	0.35	Med	14	3.2	0.48	Med	231	3.6	0.44	Med	14	3.2	0.48	Med
		1EZU	841	4.9	0.32	Med	170	4.5	0.36	Acc	841	4.9	0.32	Med	1554	3.8	0.47	Med
		1F34	62	3.4	0.60	Med	2	4.3	0.51	Acc	925	2.4	0.71	Med	3	3.7	0.58	Med
		1FLE	31	5.0	0.26	Acc	3	3.7	0.44	Med	1102	3.4	0.39	Med	192	3	0.58	Med
		1GL1	73	2.6	0.74	Med	326	3.8	0.56	Med	73	2.6	0.74	Med	881	2.3	0.73	Med
		1GXD	1173	4.9	0.46	Acc					1173	4.9	0.46	Acc				
		1HIA					18	3.4	0.29	Acc					258	2.2	0.58	Med
		1JTG	1	2.6	0.60	Med	7	4.6	0.42	Acc	1	2.6	0.60	Med	1173	3.4	0.46	Med
		1MAH	1	3.1	0.57	Med	1	2.7	0.48	Med	4	1.4	0.78	Med	4	1.9	0.63	Med
		1N8O	7	3.4	0.48	Med	11	4.8	0.34	Acc	20	0.6	0.84	High	1330	4	0.45	Acc
		1OC0	1590	4.8	0.29	Acc					1590	4.8	0.29	Acc				
		1OPH	1694	3.9	0.48	Acc					1694	3.9	0.48	Acc				
		1OYV	15	4.9	0.39	Acc	7	3.6	0.49	Med	153	3.3	0.49	Med	105	2.9	0.49	Med
		1PPE	1	2.9	0.45	Med	1	2.3	0.66	Med	3	1.1	0.76	Med	3	1.3	0.74	Med
		1R0R	138	2.2	0.67	Med	39	4.3	0.54	Med	1298	2.0	0.55	Med	1164	1.4	0.79	Med
		1TMQ	16	3.6	0.52	Med	1	4.8	0.39	Acc	885	3.0	0.47	Med	515	2.4	0.60	Med
		1UDI	24	3.5	0.45	Med	1	3.1	0.44	Med	24	3.5	0.45	Med	229	2.5	0.54	Med
		1YVB	1	2.4	0.63	Med					18	2.2	0.53	Med				
		2ABZ					5	2.8	0.51	Med					5	2.7	0.51	Med
		2B42	3	4.2	0.51	Acc	1	3.9	0.53	Acc	6	0.6	0.77	High	12	2.2	0.58	Med
		2J0T					19	2.8	0.47	Med					21	2.6	0.54	Med
		2MTA	76	4.4	0.30	Acc	90	4.3	0.39	Acc	716	0.7	0.85	High	100	3.7	0.40	Med
		2O8V	29	5.0	0.27	Acc	654	3.7	0.34	Acc	852	4.0	0.55	Med	654	3.7	0.34	Acc
		2OUL	1	1.7	0.72	Med	1	4.9	0.53	Acc	1	1.7	0.72	Med	329	3	0.36	Med
		2PCC	496	2.6	0.54	Med	10	4.3	0.49	Acc	496	2.6	0.54	Med	10	4.3	0.49	Acc
		2SIC	5	1.1	0.74	Med	1	1.1	0.76	Med	5	1.1	0.74	Med	1	1.1	0.76	Med
		2SNI	177	3.8	0.46	Med	1	4.7	0.26	Acc	299	2.8	0.50	Med	403	1.3	0.74	Med
		2UUY	693	4.4	0.38	Acc	7	4.1	0.43	Acc	1946	3.1	0.49	Med	44	3	0.44	Med
		3SGQ	428	4.0	0.37	Med	110	2.6	0.53	Med	576	1.0	0.81	Med	624	2	0.71	Med
		4CPA	1	4.4	0.32	Med	1	4.8	0.34	Med	465	2.5	0.54	Med	202	2.4	0.55	Med
		7CEI	1	4.4	0.41	Acc	1	4.1	0.33	Acc	88	0.8	0.78	High	2	1.4	0.64	Med
		BOYV					220	3.6	0.51	Med					220	3.6	0.51	Med
	Medium	1ACB	126	4.4	0.23	Acc	22	3.2	0.52	Med	393	2.6	0.55	Med	49	2.6	0.55	Med
		1IJK	81	3.0	0.61	Med	88	4.8	0.38	Acc	1317	2.0	0.66	Med	142	3.4	0.43	Acc
		1JIW																
		1KKL																
		1M10																
		1NW9					321	4.9	0.42	Med					321	2.3	0.42	Med
	Hard	1F6M																
		1FQ1																
		1PXV																
		1ZLI																
		2O3B																

Figure S12: Comparison of the performance of F^2Dock 2.0 and ZDock 3.0.2 for each of the 52 enzyme-inhibitor and enzyme-substrate complexes from ZLab's benchmark 4.0 in terms of the rank, RMSD, F_{nat} and CAPRI classification of the top hit and the best hit.

References

[1] Bajaj C, Chowdhury RA, Siddavanahalli V (2011) F2Dock: Fast Fourier Protein-Protein Docking. IEEE/ACM Transactions on Computational Biology and Bioinformatics 8: 45–58.

[2] Mintseris J, Wiehe K, Pierce B, Anderson R, Chen R, et al. (2003) Protein-protein docking

Class	Difficulty	Complex	Top ranked near-native solution								Best ranked near-native solution							
			ZDock				F2Dock				ZDock				F2Dock			
			Rank	RMSD	F_nat	Verdict	Rank	RMSD	F_nat	Verdict	Rank	RMSD	F_nat	Verdict	Rank	RMSD	F_nat	Verdict
Others	Easy	1A2K	1348	4.3	0.54	Acc	44	2.4	0.48	Med	1894	3.2	0.42	Acc	44	2.4	0.48	Med
		1AK4	1090	3.5	0.52	Acc	964	4.3	0.43	Inc	1090	3.5	0.52	Acc	964	4.3	0.43	Inc
		1AKJ	546	2.9	0.48	Med	39	3.4	0.43	Med	1632	1.7	0.64	Med	39	3.4	0.43	Med
		1AZS	42	2.9	0.59	Acc					61	2.0	0.68	Med				
		1B6C	1	2.9	0.51	Med	3	4	0.43	Acc	1	2.9	0.51	Med	3	4	0.43	Acc
		1BUH	30	3.6	0.41	Med	431	4.8	0.41	Acc	1961	3.0	0.42	Med	431	4.8	0.41	Acc
		1E96	1171	3.8	0.57	Acc	278	5	0.44	Acc	1171	3.8	0.57	Acc	314	4.2	0.52	Acc
		1EFN																
		1F51	589	4.6	0.39	Acc					589	4.6	0.39	Acc				
		1FC2					1190	5	0.31	Inc					1570	4.1	0.56	Inc
		1FCC																
		1FFW	73	4.5	0.29	Acc	325	4.7	0.29	Acc	1349	4.0	0.41	Med	1291	3	0.60	Med
		1FQJ																
		1GCQ	1105	1.4	0.65	Med					1105	1.4	0.65	Med				
		1GHQ																
		1GLA	1708	3.9	0.58	Med					1708	3.9	0.58	Med				
		1GPW	3	3.6	0.56	Acc	1	3.7	0.43	Med	134	2.1	0.55	Med	5	2.6	0.65	Med
		1H9D	1006	4.5	0.37	Med					1006	4.5	0.37	Med				
		1HCF	175	4.0	0.48	Acc	1225	4.7	0.38	Inc	225	1.9	0.64	Med	1225	4.7	0.38	Inc
		1HE1	1141	4.7	0.53	Med	574	4.9	0.42	Med	1141	4.7	0.53	Med	574	4.9	0.42	Med
		1I4D	571	4.2	0.43	Acc					571	4.2	0.43	Acc				
		1J2J					182	4.6	0.45	Med					182	4.6	0.45	Med
		1JWH	7	3.6	0.51	Acc					78	1.9	0.64	Med				
		1K74	2	1.2	0.79	Med	3	3.8	0.42	Acc	2	1.2	0.79	Med	7	2.6	0.56	Med
		1KAC	592	4.5	0.39	Acc	8	4.4	0.43	Med	1527	1.9	0.68	Med	99	4.1	0.40	Acc
		1KLU	1957	3.4	0.51	Acc					1957	3.4	0.51	Acc				
		1KTZ	535	2.8	0.73	Med	98	3.9	0.65	Acc	535	2.8	0.73	Med	166	2.9	0.55	Med
		1KXP	1	1.6	0.71	Med	7	3.5	0.48	Acc	1	1.6	0.71	Med	260	2.6	0.59	Med
		1ML0	4	3.1	0.58	Med	2	4.3	0.49	Med	8	3.1	0.55	Med	123	3.2	0.48	Med
		1OFU	84	4.5	0.30	Inc					347	3.1	0.36	Acc				
		1PVH	748	4.5	0.59	Acc					1192	1.5	0.73	Med				
		1QA9																
		1RLB	3	4.6	0.55	Acc	555	5	0.39	Acc	232	3.4	0.49	Acc	555	5	0.39	Acc
		1RV6	2	1.3	0.78	Med	2	4	0.38	Med	2	1.3	0.78	Med	694	2.2	0.41	Med
		1S1Q	756	1.9	0.58	Med					1243	1.4	0.77	Med				
		1SBB																
		1T6B	58	3.6	0.52	Acc	525	4	0.54	Acc	1510	2.8	0.52	Med	752	2.7	0.56	Acc
		1US7	74	1.1	0.76	Med					74	1.1	0.76	Med				
		1WDW	2	1.2	0.76	Med	1	2.5	0.59	Med	2	1.2	0.76	Med	1	2.5	0.59	Med
		1XD3	8	4.0	0.52	Med	1	4.2	0.47	Med	86	2.6	0.51	Med	1298	3.9	0.49	Med
		1XU1	912	5.0	0.33	Acc					912	5.0	0.33	Acc				
		1Z0K	8	3.3	0.49	Med	307	3.3	0.56	Med	8	3.3	0.49	Med	307	3.3	0.56	Med
		1Z5Y	20	3.4	0.45	Acc					423	2.5	0.63	Med				
		1ZHH																
		1ZHI	65	4.4	0.40	Acc	202	4	0.45	Med	324	2.1	0.59	Med	202	4	0.45	Med
		2A5T					268	3.6	0.50	Med					618	2.9	0.51	Acc
		2A9K					558	3.4	0.46	Acc					558	3.4	0.46	Acc
		2AJF	475	3.6	0.47	Med					475	3.6	0.47	Med				
		2AYO	37	3.3	0.47	Med	1108	2	0.57	Med	138	2.5	0.57	Med	1108	2	0.57	Med
		2B4J																
		2BTF	53	4.7	0.42	Med	95	4.5	0.35	Med	148	3.8	0.44	Med	377	3.4	0.50	Med
		2FJU	261	3.2	0.68	Acc	228	4.2	0.45	Acc	261	3.2	0.68	Acc	333	3.5	0.53	Acc

Figure S13: Comparison of the performance of F^2Dock 2.0 and ZDock 3.0.2 for each of the 99 other type of complexes from ZLab's benchmark 4.0 in terms of the rank, RMSD, F_{nat} and CAPRI classification of the top hit and the best hit. Continued as Figure S14.

benchmark 2.0: an update. Proteins 60: 214–6.

[3] Gabb HA, Jackson RM, Sternberg MJE (1997) Modelling protein docking using shape complementarity, electrostatics and biochemical information. Journal of Molecular Biology 272: 106–120.

Class	Difficulty	Complex	Top ranked near-native solution								Best ranked near-native solution							
			ZDock				F2Dock				ZDock				F2Dock			
			Rank	RMSD	F_nat	Verdict	Rank	RMSD	F_nat	Verdict	Rank	RMSD	F_nat	Verdict	Rank	RMSD	F_nat	Verdict
Others Cont.	Easy Cont	2G77	15	1.5	0.64	Med	8	3.7	0.34	Med	15	1.5	0.64	Med	917	1.3	0.74	Med
		2HLE	31	4.1	0.39	Med	4	3.8	0.47	Med	31	4.1	0.39	Med	4	3.8	0.47	Med
		2HQS					27	4.1	0.32	Acc					125	2.7	0.48	Med
		2OOB																
		2OOR	766	4.4	0.39	Acc	16	4	0.47	Med	766	4.4	0.39	Acc	63	2	0.68	Med
		2VDB	5	1.2	0.82	Med					5	1.2	0.82	Med				
		3BP8					474	5	0.26	Acc					699	3.3	0.60	Acc
		3D5S	71	3.1	0.45	Med	1	3.2	0.43	Med	609	2.5	0.61	Med	4	2.7	0.50	Med
	Medium	1GP2	61	4.4	0.42	Inc	193	4	0.63	Acc	107	2.8	0.52	Acc	193	4	0.63	Acc
		1GRN	1299	4.3	0.51	Acc	401	4.8	0.37	Acc	1299	4.3	0.51	Acc	401	4.8	0.37	Acc
		1HE8																
		1I2M	267	2.2	0.46	Med	545	2.6	0.66	Med	267	2.2	0.46	Med	545	2.6	0.66	Med
		1IB1																
		1K5D					521	4.3	0.44	Acc					521	4.3	0.44	Acc
		1LFD	85	4.6	0.34	Med	990	4.6	0.35	Acc	466	4.5	0.30	Med	1235	4.1	0.40	Acc
		1MQ8	1455	3.2	0.60	Med					1455	3.2	0.60	Med				
		1N2C																
		1R6Q					180	3.7	0.31	Med					311	3.5	0.41	Med
		1SYX	211	4.8	0.42	Acc	2	4.7	0.45	Med	211	4.8	0.42	Acc	11	3	0.55	Med
		1WQ1	81	4.0	0.45	Acc					81	4.0	0.45	Acc				
		1XQS	19	3.8	0.38	Acc	61	4.2	0.33	Acc	45	2.6	0.48	Acc	833	3.7	0.38	Med
		1ZM4	6	4.1	0.46	Acc					631	2.7	0.67	Acc				
		2CFH	1	3.8	0.55	Med	119	2.6	0.51	Med	2	1.7	0.66	Med	119	2.6	0.51	Med
		2H7V	1112	4.6	0.46	Acc					1112	4.6	0.46	Acc				
		2HRK	3	3.7	0.55	Acc					3	3.7	0.55	Acc				
		2J7P																
		2NZ8	64	4.5	0.29	Acc					64	4.5	0.29	Acc				
		2OZA																
		2Z0E					169	3.9	0.43	Med					169	3.9	0.43	Med
		3CPH					250	4.3	0.38	Acc					250	4.3	0.38	Acc
	Hard	1ATN					1307	2.7	0.46	Med					1307	2.7	0.46	Med
		1BKD																
		1DE4	84	4.7	0.43	Inc					84	4.7	0.43	Inc				
		1EER																
		1FAK																
		1H1V																
		1IBR																
		1IRA																
		1JK9	510	4.2	0.40	Acc	422	2.5	0.72	Med	790	4.1	0.49	Acc	422	2.5	0.72	Med
		1JMO																
		1JZD	44	4.6	0.37	Med	144	4	0.44	Med	44	4.6	0.37	Med	144	4	0.44	Med
		1R8S																
		1Y64																
		2C0L																
		2I9B																
		2IDO	130	3.6	0.50	Med	156	4.5	0.33	Acc	154	3.5	0.43	Med	156	4.5	0.33	Acc
		2OT3	121	4.6	0.43	Acc					327	4.5	0.28	Acc				

Figure S14: Comparison of the performance of F^2Dock 2.0 and ZDock 3.0.2 for each of the 99 other type of complexes complexes from ZLab's benchmark 4.0 in terms of the rank, RMSD, F_{nat} and CAPRI classification of the top hit and the best hit.

[4] Dolinsky TJ, Nielsen JE, McCammon JA, Baker NA (2004) PDB2PQR: An automated pipeline for the setup, execution, and analysis of Poisson-Boltzmann electrostatics calculations. Nucleic Acids Research 32: w665–w667.

[5] Jones S, Thornton JM (1997) Analysis of protein-protein interaction sites using surface patches. Journal of Molecular Biology 272: 121–132.

[6] Jones S, Thornton JM (1996) Principles of protein-protein interactions. Proceedings of the National Academy of Sciences of the United States of America 93: 13–20.

[7] Black S, Mould D (1991) Development of hydrophobicity parameters to analyze proteins which bear post- or cotranslational modifications. Analytical Biochemistry 193: 72–82.

[8] Weiner SJ, Kollman PA, Case DA, Singh UC, Ghio C, et al. (1984) A new force field for molecular mechanical simulation of nucleic acids and proteins. Journal of the American Chemical Society 106: 765–784.

[9] Morris GM, Goodsell DS, Huey R, Olson AJ (1996) Distributed automated docking of flexible ligands to proteins: Parallel applications of AutoDock 2.4. Journal of Computer-Aided Molecular Design 10: 293–304.

[10] Chowdhury RA, Bajaj C (2010) Algorithms for faster molecular energetics, forces and interfaces. ICES report 10–32, Institute for Computational Engineering & Science, The University of Texas at Austin.

[11] Bajaj C, Zhao W (2010) Fast molecular solvation energetics and forces computation. SIAM Journal on Scientific Computing 31: 4524–4552.

[12] Jackins CL, Tanimoto SL (1980) Oct-trees and their use in representing three-dimensional objects. Computer Graphics and Image Processing 14: 249–270.

[13] Bajaj C, Chowdhury RA, Rasheed M (2010) A dynamic data structure for flexible molecular maintenance and informatics. Bioinformatics 27: 55–62.

[14] Glaser F, Steinberg DM, Vakser IA, Ben-Tal N (2001) Residue frequencies and pairing preferences at protein-protein interfaces. Proteins: Structure, Function, and Genetics 43: 89–102.

[15] Martin A (2010) Protein sequence and structure analysis of antibody variable domains. Antibody engineering : 33–51.

[16] Eisenberg D, Mclachlan A (1986) Solvation energy in protein folding and binding. Nature 319: 199–203.

[17] Gilson M, Davis M, Luty B, McCammon J (1993) Computation of electrostatic forces on solvated molecules using the Poisson-Boltzmann equation. Journal of Physical Chemistry 97: 3591–3600.

[18] Hermann RB (1972) Theory of hydrophobic bonding. ii. correlation of hydrocarbon solubility in water with solvent cavity surface area. Journal of Physical Chemistry 76: 2754–2759.

[19] Simonson T, Bruenger AT (1994) Solvation free energies estimated from macroscopic continuum theory: An accuracy assessment. Journal of Physical Chemistry 98: 4683 – 4694.

[20] Wagoner J, Baker N (2006) Assessing implicit models for nonpolar mean solvation forces: The importance of dispersion and volume terms. Proceesings of the National Academy of Sciences of the USA 103: 8331–8336.

[21] Still WC, Tempczyk A, Hawley RC, Hendrickson T (1990) Semianalytical treatment of solvation for molecular mechanics and dynamics. Journal of the American Chemical Society 112: 6127–6129.

[22] Grycuk T (2003) Deficiency of the coulomb-field approximation in the generalized born model: An improved formula for born radii evaluation. Journal of Chemical Physics 119: 4817–4826.

[23] Tjong H, Zhou HX (2007) Gbr^6: A parameterization-free, accurate, analytical generalized born method. Journal of Physical Chemistry B 111: 3055–3061.
